# Comparative yield of molecular diagnostic algorithms for autism spectrum disorder diagnosis in India: evidence supporting whole exome sequencing as first tier test

**DOI:** 10.1186/s12883-023-03341-0

**Published:** 2023-08-05

**Authors:** Frenny Sheth, Jhanvi Shah, Deepika Jain, Siddharth Shah, Harshkumar Patel, Ketan Patel, Dhaval I Solanki, Anand S Iyer, Bhargavi Menghani, Priti Mhatre, Sanjiv Mehta, Shruti Bajaj, Vishal Patel, Manoj Pandya, Deepak Dhami, Darshan Patel, Jayesh Sheth, Harsh Sheth

**Affiliations:** 1 FRIGE’s Institute of Human Genetics, Ahmedabad, India; 2 Shishu Child Development and Early Intervention Centre, Ahmedabad, India; 3 Royal Institute of Child Neurosciences, Ahmedabad, India; 4 Zydus Hospital, Ahmedabad, India; 5 Specialty Homeopathic Clinic, Ahmedabad, India; 6 Mantra Child Neurology and Epilepsy Hospital, Bhavnagar, India; 7 NeuroKids Clinic, Ahmedabad, India; 8 Children’s Institute for Development and Advancement Centre, Vadodara, India; 9 Tender Kinds Centre for Child Development, Navi Mumbai, India; 10 The Purple Gene Clinic, Mumbai, India; 11 Little Brain Pediatric Neurocare Centre, Vadodara, India; 12 Kadam Maternity Home, Ahmedabad, India; 13 Axon Child Neurology and Epilepsy Centre, Rajkot, India; 14 Charotar Institute of Paramedical Sciences, Charotar University of Science and Technology, Changa, India

**Keywords:** Autism spectrum disorder, Genetic etiology, Diagnostic yield, Chromosomal microarray, Whole exome sequencing, *De novo*, Rett syndrome, India

## Abstract

**Background:**

Autism spectrum disorder (ASD) affects 1 in 100 children globally with a rapidly increasing prevalence. To the best of our knowledge, no data exists on the genetic architecture of ASD in India. This study aimed to identify the genetic architecture of ASD in India and to assess the use of whole exome sequencing (WES) as a first-tier test instead of chromosomal microarray (CMA) for genetic diagnosis.

**Methods:**

Between 2020 and 2022, 101 patient-parent trios of Indian origin diagnosed with ASD according to the Diagnostic and Statistical Manual, 5th edition, were recruited. All probands underwent a sequential genetic testing pathway consisting of karyotyping, Fragile-X testing (in male probands only), CMA and WES. Candidate variant validation and parental segregation analysis was performed using orthogonal methods.

**Results:**

Of 101 trios, no probands were identified with a gross chromosomal anomaly or Fragile-X. Three (2.9%) and 30 (29.7%) trios received a confirmed genetic diagnosis from CMA and WES, respectively. Amongst diagnosis from WES, SNVs were detected in 27 cases (90%) and CNVs in 3 cases (10%), including the 3 CNVs detected from CMA. Segregation analysis showed 66.6% (n = 3 for CNVs and n = 17 for SNVs) and 16.6% (n = 5) of the cases had *de novo* and recessive variants respectively, which is in concordance with the distribution of variant types and mode of inheritance observed in ASD patients of non-Hispanic white/ European ethnicity. *MECP2* gene was the most recurrently mutated gene (n = 6; 20%) in the present cohort. Majority of the affected genes identified in the study cohort are involved in synaptic formation, transcription and its regulation, ubiquitination and chromatin remodeling.

**Conclusions:**

Our study suggests *de novo* variants as a major cause of ASD in the Indian population, with Rett syndrome as the most commonly detected disorder. Furthermore, we provide evidence of a significant difference in the diagnostic yield between CMA (3%) and WES (30%) which supports the implementation of WES as a first-tier test for genetic diagnosis of ASD in India.

**Supplementary Information:**

The online version contains supplementary material available at 10.1186/s12883-023-03341-0.

## Background

Autism spectrum disorder (ASD) is a heterogeneous group of neurodevelopmental disorders (NDD) with a prevalence of approximately 1 in 160 children worldwide [1] and with variable clinical presentations and outcomes [2]. According to the latest version of the Diagnostic and Statistical Manual of Mental Disorders (DSM-5), it is characterized by impaired social communication along with repetitive behavior or restricted interests which can persist throughout lifetime [3, 4]. In addition to these core features, many affected individuals can be afflicted with comorbidities like intellectual disability and epilepsy. A review and meta-analysis of ASD in India reported low prevalence of only 0.0014 − 0.0012% in children aged 1–18 years compared to developed countries like the United States and United Kingdom with a prevalence of 1-1.5% [5]. However, a review across the South Asian population reported its prevalence rate ranging from 0.09 to 1.07% which is similar to that observed in developed countries [6].

The etiology of ASD is not fully understood, although, similar to several neurodevelopmental disorders, genetic risk and environmental exposure appears to contribute to the pathogenesis of ASD [7, 8]. Data from twin studies suggest a strong genetic role and a quantitative meta-analysis on all published twin studies in the context of ASD has estimated heritability component between 64 and 91% [9]. Therefore, genetic testing is recommended in ASD patients and as of 2013, an etiology underlying ASD could be established in around 6–15% cases [10]. Guidelines put forth a decade ago by the American College of Medical Genetics (ACMG) suggests using chromosomal microarray (CMA) as a first line test in ASD since its diagnostic yield was estimated to be between 7 and 9% [2, 10]. However, since then, studies using whole exome sequencing (WES) have evidenced sequence level contribution of *de novo* variants in the etiology of ASD and recent advancements in computational analyses of WES data suggests improvement in detection of copy number variants (CNVs) too. Indeed, two recent studies have shown that WES was able to detect nearly all clinically relevant CNVs that were detected by CMA thereby increasing its diagnostic yield by approximately 1.6% [11, 12]. In addition, a recent retrospective study using WES on clinically diagnosed 343 children with ASD from Spain suggested a diagnostic yield of ~ 14% with 75% of the cases harbouring a *de novo* variant [1]. It is predicted that nearly 85% of the disease causing variants reside in the protein coding and splice site regions of the genome, which are well covered by WES [13–15]. Various studies have repeatedly shown a better yield and utility of WES over CMA in NDD and thus, WES has now been suggested as a first-tier test for patients with intellectual disability/ NDD [16, 17].

Selection and availability of a first-tier test with high diagnostic yield is desirable in low-middle income countries (LMICs) like India, since patients and families bear the cost of genetic testing. To our knowledge, no study to date has been performed in the Indian population to delineate the genetic architecture of ASD which can aid in the selection of first-tier genetic test. Here, we report the first systematic study to assess the genetic architecture and molecular diagnostic yields for karyotype, Fragile-X testing, CMA and WES in a population-based cohort of 101 patient-parent trios with ASD from India.

## Materials and methods

### Patient recruitment and sample collection

The study included consecutively recruited 101 children with a confirmed clinical diagnosis of idiopathic ASD based on the DSM-5 [3, 4]. Children with prominent syndromic features, isolated speech delay or isolated sensory processing disorders were excluded from this study. Blood samples of the patient-parent trios were collected. The parents or guardians of all probands provided a written informed consent as per the Helsinki Declaration and the study was approved by the research ethics committee at Foundation for Research in Genetics and Endocrinology, Ahmedabad (ID: FRIGE/IEC/19/2020). All the methods in the study were carried out as per the Helsinki Declaration. High molecular weight genomic DNA was extracted using desalting method [18] and was stored at -20 °C until molecular genetic testing was carried out.

### Karyotyping and Fragile-X testing

Karyotyping was performed in all cases regardless of sex, whereas Fragile-X testing was performed only in male probands. Karyotyping was carried out using GTG banding at 500 band resolution to check for gross chromosomal aberrations. Fragile-X testing was carried out by triplet repeat primed – polymerase chain reaction (TP-PCR), that involved analyzing CGG repeat expansion in the 5’ UTR of the *FMR1* gene using method as previously described [19]. Children with a normal chromosomal constitution and showing no expansion of the CGG repeats in the 5’ UTR of *FMR1* gene were subsequently assessed with CMA and WES.

### Chromosomal microarray

CMA was carried out using CytoScan™ Optima array, GeneChip™ System 3000 and Affymetrix platform (Thermo Fisher Scientific, USA) as per the manufacturer’s instructions. Chromosome Analysis Suite Software (ChAS) (Thermo Fisher Scientific, USA) was used to carry out the analysis of the data as per the manufacturer’s recommendations which suggested a minimum resolution of 1 Mb for losses, 2 Mb for gains and 5 Mb for copy neutral loss of heterozygosity. For all candidate CNVs, variants were primarily screened for population frequency and known disease associations using publicly available databases like gnomAD database [20], DGV [21] and DECIPHER [22] and OMIM [23]. Pathogenicity of CNVs were classified in accordance with ACMG and ClinGen classification system [24]. All candidate CNVs were validated in proband and parents using SYBR Green based quantitative PCR (Q-PCR) using ABI’s StepOne Real Time PCR system (Thermo Fisher Scientific, USA) (Supplementary Table 1).

### Whole exome sequencing

Genomic DNA of the proband was subjected to selective capture and sequencing of the protein coding regions that included exons and exon-intron boundaries of genes using Agilent SureSelect v6 enrichment kit (Agilent, USA). The library prepared, was subjected to paired-end sequencing with a mean coverage of > 80-100x on the Illumina HiSeq or NovaSeq platform (Illumina, USA). Sequences obtained as FASTQ files were aligned to the human reference genome (GRCh37/hg19) using BWA MEM v0.7.12 [25]. SNVs and indels were called using GATK v4.12 Haplotype caller [26]. In addition to SNVs and small indels, copy number variants (CNVs) were detected from the data using the ExomeDepth v1.1.10 [27].

Variant annotation, filtration and prioritization was performed using Exomiser v12.1.0 [28]. Exomiser uses the hiPHIVE prioritization method that incorporates protein-protein interaction networks and multi-species ontologies along with ranking candidate genes based on the predicted variant pathogenicity associated with the phenotype. The phenotype information was coded in uniform human phenotype ontology (HPO) terminologies [29]. Common variants were filtered based on minor allele frequency in the 1000Genome Phase 3 [30] and gnomAD v2.1 [20] databases. The minor allele frequency cut off was set at 0.02 (2%). The cut-off was set assuming ASD has a global prevalence of 1:100; the frequency of major and minor alleles would be 0.9 (p) and 0.1 (q), respectively, based on the Hardy-Weinberg equilibrium. As ASD is caused by dominant *de novo* variants in majority of the cases (pq = 0.09) and the prior estimates suggests genetic diagnostic yield of approximately 33%, pq would be 0.027. Only non-synonymous variants in the coding region and canonical splice site variants with a depth of > 20x were used for analysis and clinical correlation. Various *in-silico* prediction tools such as PolyPhen-2 [31], SIFT [32], MutationTaster2 [33], LRT [34], CADD [35] and MetaDome [36] were used to predict pathogenicity of non-synonymous and indel variants. A CADD_phred score of ≥ 15, slightly intolerant, intolerant or highly intolerant predictions of MetaDome and at least two damaging predictions from the remaining *in silico* tools were used for selection of candidate variants. *In-silico* predictions along with available knowledge from various sources and databases as described below was used in prioritising the variant.

Post-gross filtering, variants were prioritized based on the following: (a) known disease causing variant previously reported in databases like ClinVar [37] and HGMD [38]; (b) novel variants in known genes based on the Z-score for missense and pLoF or LOEUF score for loss of function variants available in the gnomAD database [20]; (c) variants in novel candidate genes wherein the respective gene was additionally evaluated for their function using UniProt [39] and Human Protein Atlas (proteinatlas.org) [40]. Tissue expression using GTEx database (gtexportal.org), association/ interaction with known ASD genes using STRING database [41] and, plausible phenotypic outcome in murine models based on the MGI database [42] were assessed. All candidate variants were assessed using IGV [43] to evaluate their quality.

In the case of candidate CNVs, variants were primarily screened for population frequency and known disease associations using publicly available databases like gnomAD database [20], DGV [21], DECIPHER [22] and OMIM [23]. Pathogenicity of CNVs were classified in accordance with ACMG and ClinGen classification system [24].

All candidate SNVs and indels were validated in proband and parents using bi-directional Sanger sequencing using ABI’s SeqStudio platform (Thermo Fisher Scientific, USA) whereas all candidate CNVs were validated using SYBR Green based quantitative PCR (Q-PCR) using ABI’s StepOne Real Time PCR system (Thermo Fisher Scientific, USA) (Supplementary Table 1). This was conducted to delineate mode of inheritance and reclassify variant pathogenicity.

The classification of SNVs was carried out according to the American College of Medical Genetics – American College of Pathologists (ACMG-AMP) guidelines [44] and ClinGen framework [24].

## Results

### Study cohort

The study cohort consisted of 101 well defined patient-parent trios diagnosed with moderate to severe ASD of unknown etiology as per the DSM-5 criteria. The average age at recruitment was 5 ± 3 years and ranged from 2 to 6 months to 16 years (Table 1). The average maternal and paternal age at the time of conception was 28 ± 4 years and 30 ± 4 years, respectively. The cohort included 72 males (71%) and 29 females (29%), suggesting a male to female ratio of approximately 3:1. Five families had more than one child diagnosed with ASD (Supplementary Information 1). Consanguinity was noted in 8 families (7.9%), whereas non-consanguinity and endogamy in 31 (30.7%) and 62 (61.4%) families, respectively. All 101 probands with ASD also had developmental delay and intellectual disability with some of them having subtle dysmorphism (large and/ or cupped ears, long eyelashes, telecanthus, thin upper lip) (n = 28/101; 27.7%) and epilepsy (n = 28/101; 27.7%) (Supplementary Table 2).

**Table 1:** Demographics of 101 patient-parent trios

Variable	Whole Sample (N = 101)
**Gender, n (%)**	
Male	73 (72)
Female	28 (28)
Male-female ratio	2.6:1
**Age, years (SD)**	
Age at diagnosis of probands	5 (3)
Maternal age at conception	28 (4)
Paternal age at conception	30 (4)
**Type of Marriage, n (%)**	
Consanguineous	8 (8)
Non-consanguineous	31 (31)
Endogamous	62 (61)
**Phenotype, n (%)**	
Developmental delay	101 (100)
Speech delay	101 (100)
Intellectual disability	101 (100)
Epilepsy/ seizures	28 (27.7)
Subtle facial dysmorphism	28 (27.7)
Regression (social/ speech)	55 (54.4)
**Genetic testing received**^**a**^, **n (%)**	
Karyotype	101 (100)
Fragile-X (*FMR1* triplet repeat expansion)	73 (72)
Chromosomal microarray	101 (100)
Whole exome sequencing ^b^	99 (98)

^a^ Genetic testing was carried out in proband only. In cases with a candidate variant, orthogonal testing approaches (Sanger sequencing and/or Q-PCR) were used to assess and validate the variant in the parents

^b^ Whole exome sequencing was carried out in 99 of 101 cases, as the cohort contained two monozygotic twin pairs and only one proband from each twin pair was processed

### Outcomes from karyotype and fragile X testing

Sequential genetic testing was performed in all 101 patients which began with karyotyping and were followed by fragile X testing (only in male probands), CMA and WES. None of the probands showed gross chromosomal aberrations or had expanded triplet repeat tracks (full-mutation alleles with > 200 CGG repeats) in the 5’-UTR region of the *FMR1* gene. Therefore, all probands were subsequently tested using CMA and WES.

### Outcomes from chromosomal microarray

From the 101 probands in whom CMA was performed, pathogenic CNVs were detected in 3 cases (2.9%) including two deletions and one duplication (Table 2). Proband ASD-076 had an 8 Mb deletion at the 15q11.2 locus which encompassed 20 OMIM genes and is known to cause 15q11.2 deletion syndrome (OMIM#615,656) or Angelman syndrome (OMIM#105,830). Compared to the individuals with class II deletions (BP2-BP3; ISCA-37,478), individuals with large class I deletions (BP1-BP3; ISCA-37,404) at the 15q11.2 region are observed to have a high likelihood of language impairment and autistic traits, similar to that seen in the proband in our study [45]. Patient ASD-103 was detected with a deletion of 0.19 Mb size at the 9q34.3 locus which encompassed 6 OMIM genes and is associated with Kleefstra syndrome I (OMIM#610,253). Individuals with > 1 Mb deletion of the 9q34 locus have a severe phenotype such as congenital anomalies including heart defects, limb anomalies, seizures and respiratory distress. In contrast individuals having < 1 Mb deletion are observed with a milder phenotype, which in part could explain the phenotype in the proband in the current study such as bruxism, drooling, subtle facial dysmorphism and recurrent episodes of vomiting [46, 47]. Lastly, proband ASD-050 was detected with a 0.52 Mb duplication on the 1q22 locus which consists of 8 OMIM genes. This is a rare CNV which has previously only been reported in a boy with intellectual disability and psychiatric disturbances [48]. Multiple individuals in this family were affected and the duplication variant segregated with the neurological features in all family members with this variant. All CNVs in our cohort were *de novo* in origin and were observed exclusively in male probands.

**Table 2:** List of cases observed with pathogenic or likely pathogenic copy number variation using CMA and/or WES.

Sr No	Case ID	Chr No	Chromosomal microarray		Whole exome sequencing		CNV Type	Zygosity	Inheritance pattern	Inherited from	Variant classification	OMIM Disease (OMIM ID)
			Genomic coordinate	Size (Kbp)	Genomic coordinate	Variant						
1	ASD-021	15	-	-	?_20279345_21145729_?	chr15:g.(?_20279345)_(21145729_?)del	loss	Het	AD	*De novo*	Variant of uncertain significance	Chromosome 15q11.2 deletion syndrome (#615,656)
2	ASD-050	1	arr[GRCh37] 1q22(155446632_155971760)x3	525	?_155611966_156081998_?	chr1:g.(?_155611966)_(156081998_?)dup	gain	Het	AD	*De novo*	Likely pathogenic	Chromosome 1q22 microduplication syndrome
3	ASD-076	15	arr[GRCh37]15q11.2q13.2(22770421_30913574)x1	8,143	?_24955049_28272386_?	chr15:(?_24955049)_(28272386_?)del	loss	Het	AD	*De novo*	Pathogenic	Chromosome 15q11.2 deletion syndrome (#615,656)
4	ASD-103	9	arr[GRCh37]9q34.3(140338142_140533378)x1	195	?_137426248_137629023_?	chr9:g.(?_137426248)_(137629023_?)del	loss	Het	AD	*De novo*	Pathogenic	Kleefstra syndrome-1 (#610,253)

Chr = chromosome; No = number; Kbp = kilo basepair; CNV = copy number variation; Het = heterozygous; AD = autosomal dominant

### Outcomes from whole exome sequencing

WES was carried out in 99 of 101 cases, as the cohort contained two monozygotic twin pairs and only one proband from each twin pair was processed for WES. The 99 cases also included the three cases that yielded a result by CMA to assess the sensitivity of WES to detect CNVs. On an average, approximately 3 candidate gene(s) or variant(s) were identified per proband (Supplementary Table 3).

From the 101 patients, pathogenic and/ or likely pathogenic variants were identified in 30 cases (29.7%), of which, SNVs were detected in 27 cases (90%) and CNVs in 3 cases (10%) (Table 3). Interestingly, 3 CNVs detected by CMA were also identified by WES, however, a 0.8 Mb *de novo* deletion encompassing the BP1 region of the 15q11.2 locus was detected by WES alone (Table 2). On further analysis, the lack of detection of the aforementioned CNV by CMA was due to the lack of probes covering this region on CytoScan™ Optima array.

**Table 3:** List of cases observed with pathogenic or likely pathogenic single nucleotide variation using WES

Sr No	Case ID	Chr No	Genomic coordinate	Ref allele	Alt allele	Gene (OMIM ID)	Transcript ID	Exon	Variant	Protein change	Zygosity	Mode of inheritance	Inherited from	Variant classification	OMIM Disease (OMIM ID)
1	ASD-001	20	62,071,010	C	A	*KCNQ2* (*602,235)	ENST00000359125.2 NM_172107.4	6	c.868G > T	p.Gly290Cys	Het	AD	*De novo*	Pathogenic	Developmental and epileptic encephalopathy 7 (#613,720);
2	ASD-003	7	114,299,416	G	A	*FOXP2* (*605,317)	ENST00000408937.3 NM_148898.4	13	c.1549G > A	p.Ala517Thr	Het	AD	not Maternal ^a^	Likely pathogenic	Speech-language disorder-1 (#602,081)
3	ASD-010	2	166,172,149	G	T	*SCN2A* (*182,390)	ENST00000375437.2 NM_001040142.2	11	c.1552G > T	p.Glu518Ter	Het	AD	*De novo*	Pathogenic	Developmental and epileptic encephalopathy 11 (#613,721)
4	ASD-011	1	19,212,049	-	GGTCTGCA	*ALDH4A1* (*606,811)	ENST00000375341.3 NM_003748.4	5	c.363_370dup	p.Arg124LeufsTer9	Comp het	AR	Maternal	Pathogenic	Hyperprolinemia, Type II (#239,510)
			19,209,638	C	G			7	c.658G > C	p.Ala220Pro			Paternal	Likely pathogenic	
5	ASD-013	17	28,530,262	T	TC	*SLC6A4* (*182,138)	ENST00000261707.3 NM_001045.6	14	c.1745dup	p.Thr583AsnfsTer23	Het	AD; -	Maternal	Likely pathogenic	{Obsessive-compulsive disorder} (#164,230); {Anxiety-related personality traits} (#607,834)
6	ASD-016	X	153,363,096	CGGCG	-	*MECP2* (*300,005)	ENST00000453960.2 NM_001110792.2	1	c.23_27del	p.Ala8GlufsTer32	Het	XLD	*De novo*	Pathogenic	Rett syndrome (#312,750)
7	ASD-017	3	184,072,060	G	A	*TBL1XR1* (*608,628)	ENST00000457928.2 NM_024665.7	7	c.679G > A	p.Asp227Asn	Het	AD	*De novo*	Likely pathogenic	Intellectual developmental disorder, autosomal dominant 41 (#616,944); Pierpont syndrome (#602,342)
8	ASD-020	X	153,296,473	-	C	*MECP2* (*300,005)	ENST00000453960.2 NM_001110792.2	3	c.842dup	p.Arg282ProfsTer61	Het	XLD	*De novo*	Pathogenic	Rett syndrome (#312,750)
9	ASD-022	3	184,072,060	G	A	*CLCN2* (*600,570)	ENST00000265593.4 NM_004366.6	15	c.1550 C > T	p.Thr517Met	Het	AD	Paternal	Likely pathogenic	{Epilepsy, idiopathic generalized, susceptibility to, 11} (#607,628)
10	ASD-034	5	134,871,150	-		*NEUROG1* (*601,726)	ENST00000314744.4 NM_006161.3	1	c.228_231dup	p.Thr78Profs*122	Homo	UNK	Maternal and Paternal	Likely pathogenic	-
11	ASD-045	19	39,805,801	A	G	*LRFN1* (*612,807)	ENST00000248668.4 NM_020862.2	1	c.176T > C	p.Val59Ala	Het	UNK	*De novo*	Likely pathogenic	-
12	ASD-046	7	147,092,836	C	T	*CNTNAP2* (*604,569)	ENST00000361727.3 NM_014141.6	10	c.1634 C > T	p.Ala545Val	Het	AD	*De novo*	Likely pathogenic	{Autism susceptibility 15} (#612,100)
13	ASD-047	18	52,896,081	G	A	*TCF4* (*602,272)	ENST00000398339.1 NM_001243226.3	19	c.2182 C > T	p.Arg278Ter	Het	AD	*De novo*	Pathogenic	Pitt-Hopkins syndrome (#610,954)
14	ASD-049	2	166,245,627	T	A	*SCN2A* (*182,390)	ENST00000375437.2 NM_001040142.2	27	c.5311T > A	p.Tyr1771Asn	Het	AD	*De novo*	Likely pathogenic	Developmental and epileptic encephalopathy-11; (#613,721); Benign familial neonatal-infantile seizures-3; (#607,745); Episodic ataxia type 9 (618,924)
15	ASD-053	5	149,629,805	G	T	*CAMK2A* (*114,078)	ENST00000348628.6 NM_015981.4	11	c.884 C > A	p.Ala295Asp	Het	AD	*De novo*	Likely pathogenic	Intellectual developmental disorder, autosomal dominant 53 (#617,798)
16	ASD-054	X	44,949,169	C	T	*KDM6A* (*300,128)	ENST00000377967.4 NM_021140.4	25	c.3730 C > T	p.Leu1244Phe	Hemi	XL	Maternal	Likely pathogenic	Kabuki syndrome 2 (#300,867)
17	ASD-057	X	153,296,824	G	C	*MECP2* (*300,005)	ENST00000453960.2 NM_001110792.2	3	c.491 C > G	p.Pro164Arg	Het	XLD	*De novo*	Pathogenic	Rett syndrome (#312,750)
18	ASD-058	17	60,042,409	G	T	*MED13* (*603,808)	ENST00000397786.2 NM_005121.3	20	c.4802 C > A	p.Ser1601Ter	Het	AD	Maternal	Pathogenic	Intellectual developmental disorder, autosomal dominant 61 (#618,009)
19	ASD-064	X	153,296,882	G	A	*MECP2* (*300,005)	ENST00000453960.2 NM_001110792.2	3	c.433 C > T	p.Arg145Cys	Het	XLD	*De novo*	Pathogenic	Rett syndrome (#312,750)
20	ASD-067	X	153,296,777	G	A	*MECP2* (*300,005)	ENST00000453960.2 NM_001110792.2	3	c.538 C > T	p.Arg180Ter	Mosaic, somatic variant	XLD	*De novo*; post-zygotic event	Pathogenic	Rett syndrome (#312,750)
21	ASD-072	X	70,389,622	T	G	*NLGN3* (*300,336)	ENST00000358741.3 NM_181303.1	8	c.2222T > G	p.Leu741Arg	Hemi	XL	*De novo*	Likely pathogenic	{Autism susceptibility, X-linked 1} (#300,425)
22	ASD-074	9	77,249,655	C	T	*RORB* (*601,972)	ENST00000376896.3 NM_006914.4	3	c.202 C > T	p.Gln68Ter	Het	AD	Paternal (affected father)	Pathogenic	{Epilepsy, idiopathic generalized, susceptibility to, 15} (#618,357)
23	ASD-080	17	29,553,492	C	T	*NF1* (*613,113)	ENST00000358273.4 NM_001042492.3	18	c.2041 C > T	p.Arg681Ter	Het	AD	*De novo*	Pathogenic	Neurofibromatosis, type 1; (#162,200) Neurofibromatosis-Noonan syndrome (#601,321)
24	ASD-082	7	70,231,162	G	A	*AUTS2* (*607,270)	ENST00000342771.4 NM_015570.4	9	c.1531G > A	p.Gly511Arg	Het	AD	*De novo*	Likely pathogenic	Intellectual developmental disorder, autosomal dominant 26 (#615,834)
25	ASD-084	2	97,377,437	C	T	*LMAN2L* (*609,552)	ENST00000377079.4 NM_001142292.2	7	c.773G > A	p.Arg258His	Hom	AR	Maternal & Paternal	Likely pathogenic	? Intellectual developmental disorder, autosomal recessive 52 (#616,887)
26	ASD-085	5	125,887,798	G	A	*ALDH7A1* (*107,323)	ENST00000409134.3 NM_001182.5	14	c.1232 C > T	p.Pro411Leu	Hom	AR	Maternal & Paternal	Likely pathogenic	Epilepsy, pyridoxine-dependent (#266,100)
27	ASD-105	X	153,296,363	G	A	*MECP2* (*300,005)	ENST00000453960.2 NM_001110792.2	3	c.952 C > T	p.Arg318Cys	Het	XL	*De novo*	Pathogenic	Autism susceptibility, X-linked 3 (#300,496)

Het: heterozygous; Hom: homozygous; Hemi: hemizygous; Comp het: Compound heterozygous; AD: autosomal dominant; AR: autosomal recessive; XL: X-linked; UNK: unknown

^a^ Paternal DNA sample was unavailable and the variant was not detected in maternal sample

Segregation analysis revealed that approximately 66.6% (n = 3 for CNVs and n = 17 for SNVs) of the cases were caused due to a *de novo* variant. *De novo* SNVs were found primarily in previously known ASD genes- *MECP2, SCN2A, KCNQ2, TBL1XR1, CNTNAP2, TCF4, CAMK2A, NF1, AUTS2, FOXP2* and *NLGN3.* Of 17 *de novo* variants, 6 were predicted to be loss of function (pLOF) variants (35.2%) whereas the remaining were missense variants. Remarkably, 6 of the 17 patients had a *de novo* SNV in the *MECP2* gene, which is associated with Rett syndrome (OMIM#312,750). Of these, 5 were female and 1 was a male proband. Interestingly, in a rare case of the male proband aged 2.5 years with Rett syndrome, we observed that the variant c.538 C > T (p.Arg180Ter) in the *MECP2* gene originated through a post-zygotic *de novo* event which led to somatic mosaicism in the proband (Table 3) [49].

In our cohort of patients with pathogenic/ likely pathogenic variants, 5 probands (n = 5/30; 16.6%) were observed with biallelic or hemizygous variants in genes associated with NDD or metabolic disorders with a recessive mode of inheritance (Table 3). Specifically, biallelic variants were detected in (i) *ALDH4A1* gene which is associated with hyperprolinemia type II (OMIM#239,510), (ii) *NEUROG1* gene which is associated with congenital cranial dysinnervation disorder and autism spectrum disorder [50], (iii) *KDM6A* gene which is associated with Kabuki syndrome 2 (OMIM#300,867), (iv) *LMAN2L* gene which is associated with mental retardation 52 (OMIM#616,887) and, (v) *ALDH7A1* gene which is associated with pyridoxine dependent epilepsy (OMIM#266,100).

In addition, 4 probands were identified with pathogenic/ likely pathogenic heterozygous variants, which were inherited from one of their parents. In 2 cases, the variants were inherited from unaffected mother and in 1 case the variant was inherited from an unaffected father. In the 4th case, pLOF variant c.202 C > T (p.Gln68Ter) in the *RORB* gene was inherited from father who also had a clinical history of seizures (Supplementary Table 2; Supplementary Information 1). Of note, in one case (ASD-003), paternal sample was un-available, hence the mode of inheritance couldn’t be deduced. Interestingly, ASD probands with epilepsy had a higher diagnostic yield (n = 15/28; 53.6%) compared to ASD probands without epilepsy (n = 15/73; 20.5%) (*χ*^2^ = 10.6, p = 0.001), however, no such association was observed for facial dysmorphism (*χ*^2^ = 0.67, p = 0.41) and social/ speech regression phenotypes (*χ*^2^ = 0.53, p = 0.47).

Lastly, WES identified 22 VUS variants in 21 patients (n = 21/101; 20.8%; Supplementary Table 4). The variants were identified in genes that have previously been associated with or implicated in ASD etiology as per the Simons Foundation Autism Research Initiative (SFARI) Gene Database and Autism Database (AutDB). Of these, majority of the probands were detected with heterozygous variants (66.6%) which were inherited from either of the unaffected parents with equal distribution. Of note, 3 of the 21 patients following segregation analysis were detected with missense variants in the *KMT2C* gene (Kleefstra syndrome 2; OMIM#617,768) which were inherited from a healthy parent. Whilst the majority of the cases have been reported with a *de novo* variant in the *KMT2C* gene, 4 reports observed variants being inherited from a healthy parent suggesting a potential oligogenic mode of inheritance [51–54].

## Discussion

Almost a decade ago, the ACMG published guidelines recommending CMA as a first tier test for delineating the genetic cause of ASD and other NDDs [2, 10]. Since then, WES coupled with advancements in computational analyses has led to simultaneous detection of SNVs and CNVs. Studies carried out in multiple ethnic populations since 2015 have shown an increased diagnostic yield from WES compared to CMA in ASD [1, 2, 55, 56]. This outcome is supported by the observation of a high proportion of *de novo* SNVs in ASD patients which are not detectable by CMA. To our knowledge, we here report the first description of the genetic architecture of ASD and simultaneously carry out diagnostic yield comparisons of karyotype, *FMR1* triplet repeat expansion, CMA and WES in a cohort of 101 patient-parent trios of Indian origin.

Our data is in congruence with prior reports and supports the utility of WES as a primary genetic diagnostic method for ASD. In the present cohort, WES detected pathogenic/ likely pathogenic variants causative of the ASD phenotype in 29.7% of the cases in contrast with 2.9%, 0% and 0% from CMA, *FMR1* triplet repeat expansion and karyotype testing, respectively. Indeed, all three CNVs detected by CMA were also detected by WES together with a fourth CNV which was detected by WES alone. Interestingly, the low yield of CMA in the present cohort can be attributed to two potential reasons. First, gross dysmorphism was an exclusion criteria during recruitment of cases for the study. Prior study by Tammimes et al., has shown a higher diagnostic yield of CMA in children with ASD and major congenital anomaly compared with children with minor physical anomaly [2]. Two, Affymetrix CytoScan Optima oligonucleotide array was used in the current study. The platform consists of 315,608 probes and requires at least 25 probes to call a loss or gain of approximately 100 kb in size. Prior study has shown a trend for differential diagnostic yield with CMA based on both platform resolution and phenotypic manifestation in ASD patients [2]. A higher resolution microarray (1 million probes or more) had a higher diagnostic yield in ASD patients with minor physical anomalies compared to low resolution microarray (44k platform), however, this difference was abated when the test was carried out in ASD patients with major congenital anomalies [2]. It is therefore plausible that the current platform may have missed CNVs that are beyond its detection limit, which could have been picked up with a higher resolution microarray platform. The diagnostic yield in the present cohort is concordant with those reported previously from individual cohort studies [1, 2, 55, 56]. Indeed, a recent meta-analysis in patients with NDD i.e. global developmental delay, intellectual disability and ASD showed diagnostic yield of WES to range from 31 to 53% in contrast to CMA with yield ranging 15–20% [16]. Based on these results, Srivastava et al. outlined a consensus statement and a stepwise algorithm for NDD diagnosis whereby WES is presented as the first-tier test followed by CMA and/or other orthogonal tests.

**Fig. 1:**
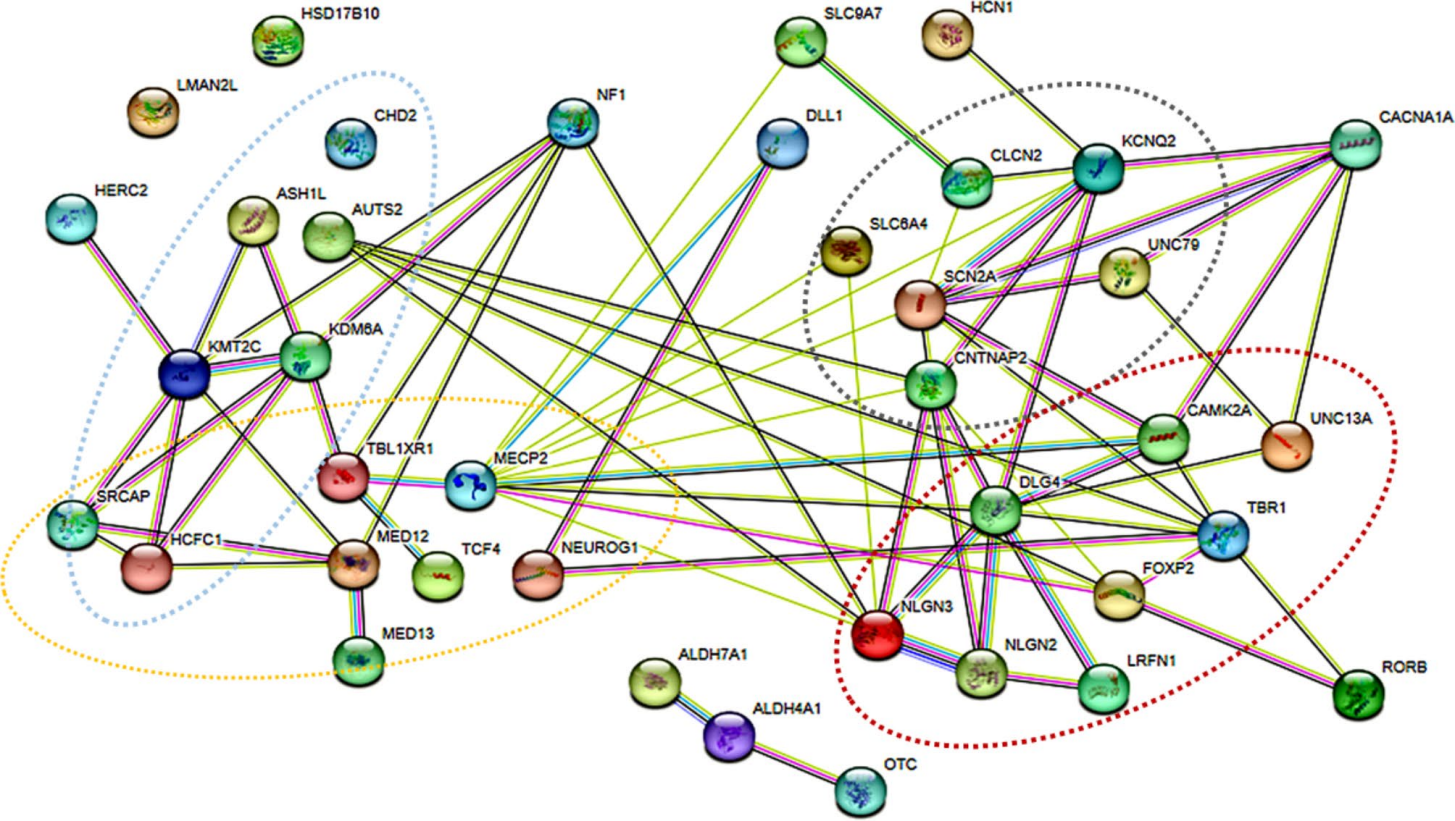
STRING network analysis show genes involved in synaptic junction formation (dark red), signal transduction (grey), transcription regulation (orange) and histone modification (light blue)

Interestingly, we observed that in 66.6% and 16.1% of the cases with a genetic diagnosis for ASD, the mode of inheritance for the variant was *de novo* and recessive, respectively. This is in congruence with prior patient-parent trio cohort studies whereby similar rates for variant’s mode of inheritance was observed [1, 2, 57]. All genes identified carrying potential causative variants were subjected to STRING analysis v11.5 (Fig. 1). The network statistics consisted of 37 unique proteins resulting in 67 various protein-protein interactions (PPI) amongst themselves. In comparison, a random set of same number of proteins, would result in only 12 different interactions. With a *p*-value of < 1.0e-16, a statistically significant enrichment of PPI in the present cohort indicated a biological connection amongst these proteins. Majority of these proteins are involved in synaptic formation, transcription and its regulation, ubiquitination and chromatin remodeling, as have been observed in prior studies [58]. This leads to a plausible hypothesis that the genetic architecture and etiopathogenesis of ASD is similar across ethnicities and an introduction of a uniform stepwise genetic testing algorithm would yield similar diagnostic yields.

In our cohort, three genes (*LRFN1*, *UNC13A* and *UNC79*) were identified as potential novel candidates for ASD. The variant in the *LRFN1* gene was a result of a *de novo* event. *LRFN1* interacts with *DLG4*, a known ASD gene vital in the formation of the post-synaptic complex required for signal transduction [59]. *DLG4* is classed under a high confidence category with a gene score of 1 in the SFARI database and has an Evaluation of Autism Gene Link Evidence (EAGLE) score of 2.45, which suggests limited but no contradicting evidence of its role in ASD. Due to the direct interaction between the two genes, *LRFN1* could be considered as a potential candidate for ASD, although functional validation is required and was beyond the scope of the current study. The variants in the *UNC13A* and *UNC79* genes were inherited from likely asymptomatic parents and classed as VUS. Both these genes have been listed in the AutDB and SFARI database and have been considered novel due to the absence of an associated phenotype in the OMIM database. A patient with developmental delay, dyskinetic movement disorder and autism has been previously identified with a *de novo* variant in the *UNC13A* gene [60]. Additionally, experimental evidence suggests its direct interaction with a known ASD associated gene, *STXBP1*. Only recently, *UNC79* gene has also been associated with neurodevelopmental features including autism [61].

With an increasing awareness of ASD amongst the general populous, there is a high likelihood of increase in demand for genetic testing in children with ASD. In a survey of parents having a child with ASD in USA, 80% of the parents indicated that they would pursue genetic testing to identify risk of ASD in the younger sibling [62]. However, financial concerns, not being offered genetic testing by a physician or a geneticist and lack of awareness are amongst the most common reasons for not opting for genetic diagnosis [63]. In addition, with the advent of development and deployment of new treatments such as trofinetide for Rett syndrome, there is likely to be increase in uptake for genetic testing [64]. This suggests that adoption of a uniform genetic testing algorithm coupled with educating primary care physicians and non-genetic specialists could improve rates of genetic testing and diagnosis in children with ASD.

### Limitations

The limitations of our study include a relatively small sample size, possible ascertainment bias related to patients having primarily non-syndromic form of ASD without gross congenital dysmorphism, carrying out WES and CMA in the proband only followed by segregation analysis by orthogonal approaches on prioritized variants and absence of detailed cost-effectiveness assessment. Despite this, we observe similar diagnostic yields to that observed in other cohorts [1, 2, 55]. Additionally, there are technical and interpretation limitations to the identification and prioritization of variants which were classified as VUS. Delineation of pathogenicity of these variants is often challenging because of their incomplete penetrance, variable expressivity and/or sex specific bias [65]. This however would require re-assessment of WES data every 2–3 years as per the consensus statement by Srivastava et al. using updated datasets and new computational tools [16]. Lastly, WES and CMA due to their inherent technical limitations are unable to resolve complex structural re-arrangements (e.g. inversions and translocations) which could play role in the pathogenesis of NDD [66], although, newer genomic technologies such as long-read whole genome sequencing could help to assess their role in the etiology of ASD.

## Conclusion

Data from large scale genomic and transcriptomic studies have helped to delineate the genetic architecture of ASD in European/ non-Hispanic white populations. To the best of our knowledge, this is the first study to delineate the genetic architecture of ASD in the Indian population, with *de novo* variants in genes involved in synaptic formation, transcription and its regulation, ubiquitination and chromatin remodeling as the primary cause. In congruence with data from other ethnic populations, the current study provides evidence supporting the implementation of WES as the first-tier test in the genetic diagnosis of ASD.

### Electronic supplementary material

Below is the link to the electronic supplementary material.

Supplementary Material 1

Supplementary Material 2

Supplementary Material 3

Supplementary Material 4

Supplementary Material 5

## Data Availability

Datasets supporting the conclusions of this article are available on the EGA website (European Genome-Phenome Archive) under the title “Genetic architecture of autism spectrum disorders in India”. To access whole exome sequencing data, the study ID is EGAS00001006060 and to access chromosomal microarray data, the study ID is EGAS00001006439. Datasets can be accessed from the EGA website using the following weblink: https://ega-archive.org/.

## Abbreviations

ASD: autism spectrum disorder
NDD: neurodevelopmental disorder
DSM-5: Diagnostic and statistical manual − 5th edition
CMA: chromosomal microarray
WES: whole exome sequencing
SNV: single nucleotide variant
CNV: copy number variant
VUS: variants of uncertain significance
PPI: protein-protein interactions
SFARI: Simons Foundation Autism Research Initiative
AutDB: Autism Database
