# Supplementary material for: Comparative yield of molecular diagnostic algorithms for autism spectrum disorder diagnosis in India: evidence supporting whole exome sequencing as first tier test
Source: BMC Neurol. 2023 Aug 5;23:292. doi: 10.1186/s12883-023-03341-0 (PMC10403833; doi:10.1186/s12883-023-03341-0)
Supplement: Supplementary file 2 — Supplementary Material 2 [file 12883_2023_3341_MOESM2_ESM.docx]

**Supplementary Information 1:** Pedigree charts of 101 ASD patient-parent trios in the current cohort.

| ASD-001  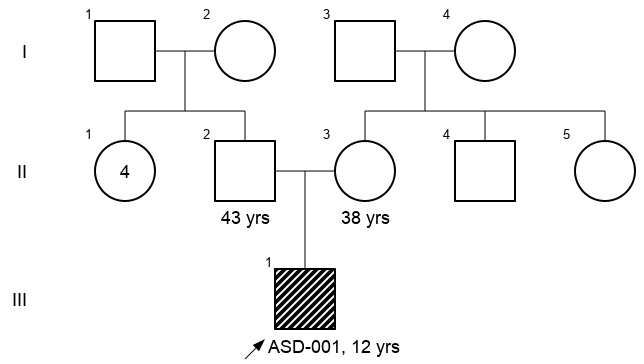 | ASD-002  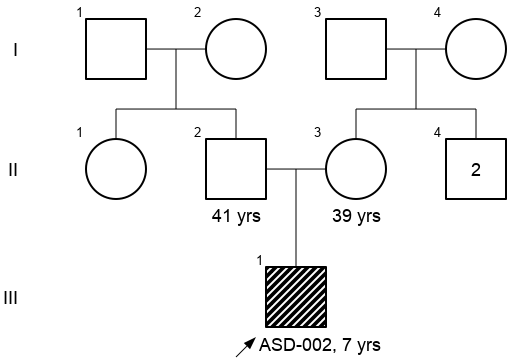 |
| --- | --- |
| ASD-003  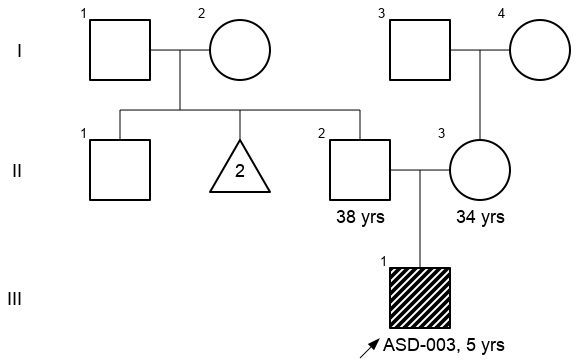 | ASD-004  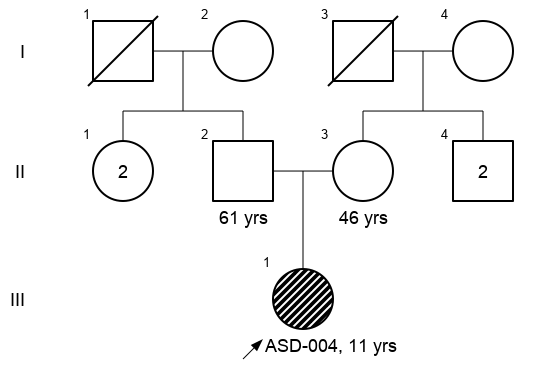 |
| ASD-005  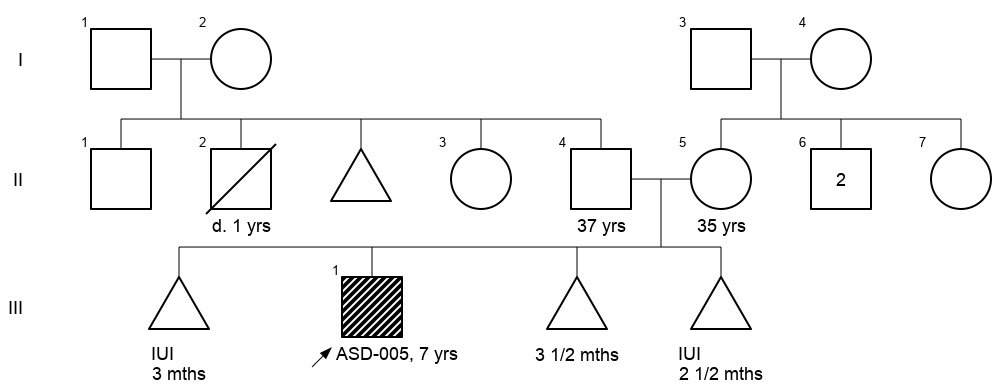 | ASD-006  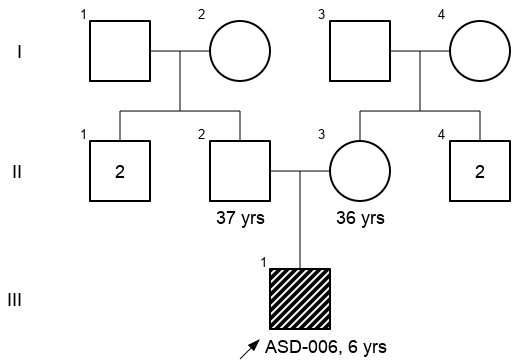 |
| ASD-007  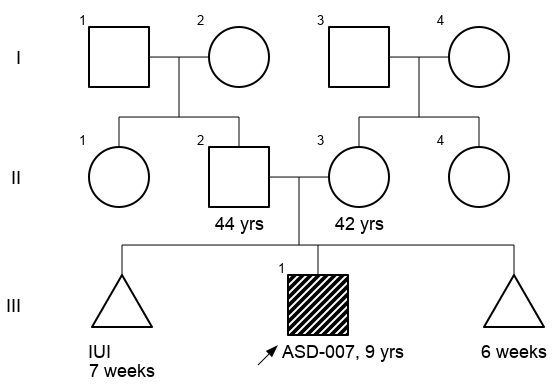 | ASD-008  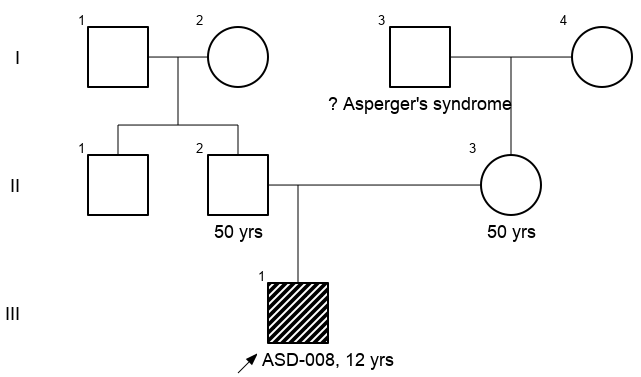 |
| ASD-009  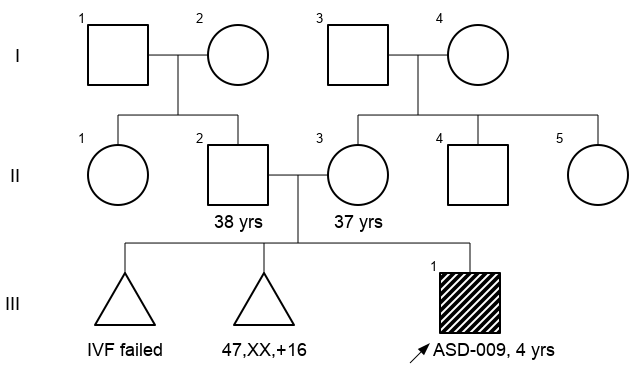 | ASD-010  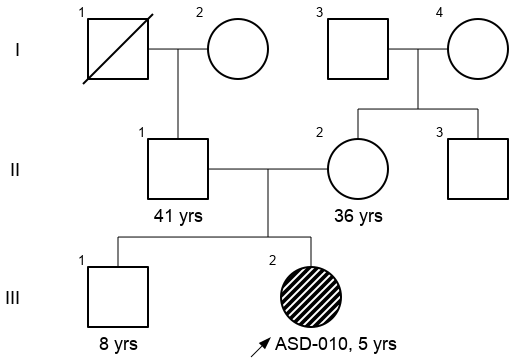 |
| ASD-011  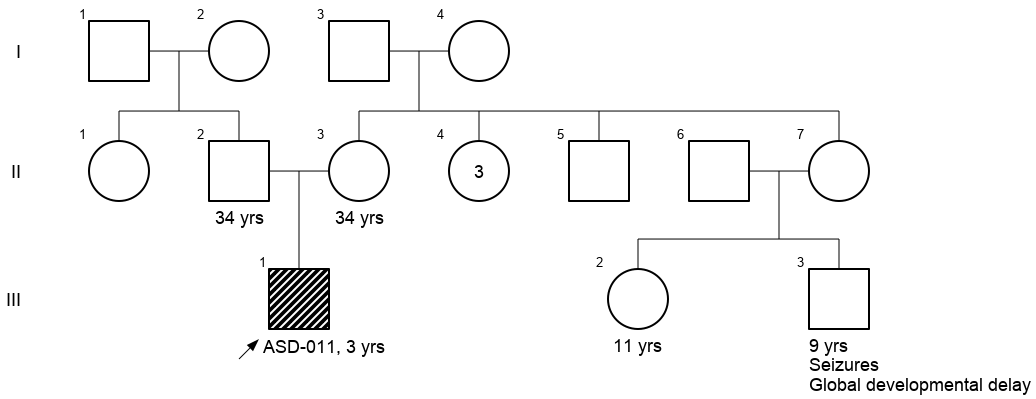 | ASD-012  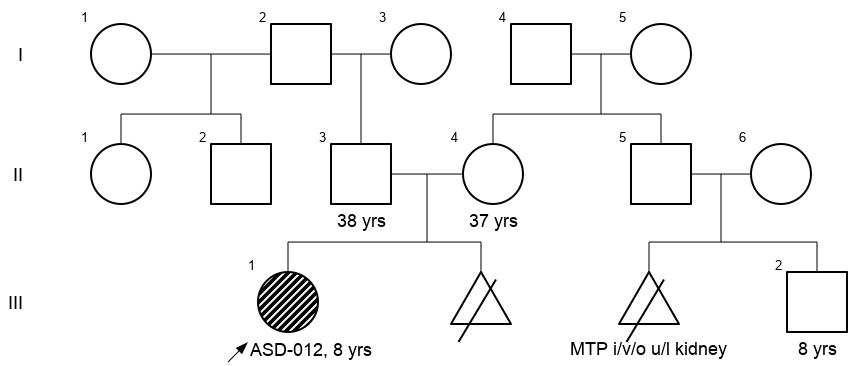 |
| ASD-013  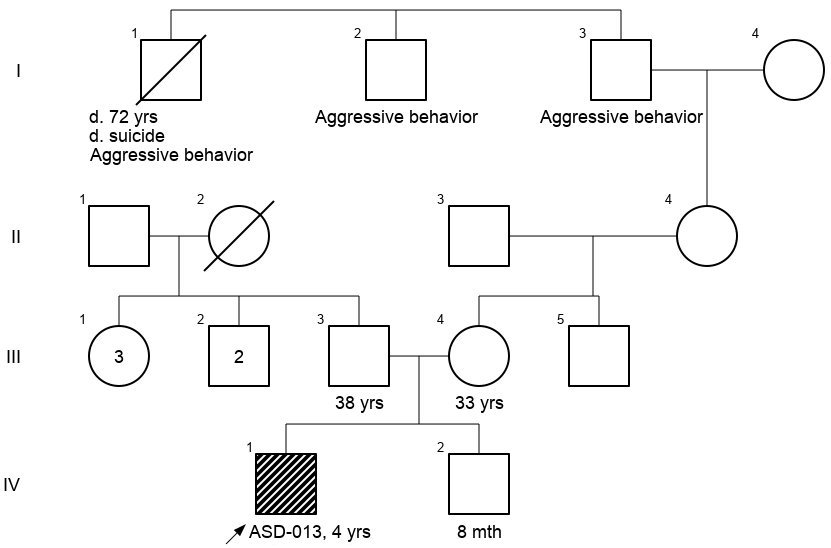 | ASD-014  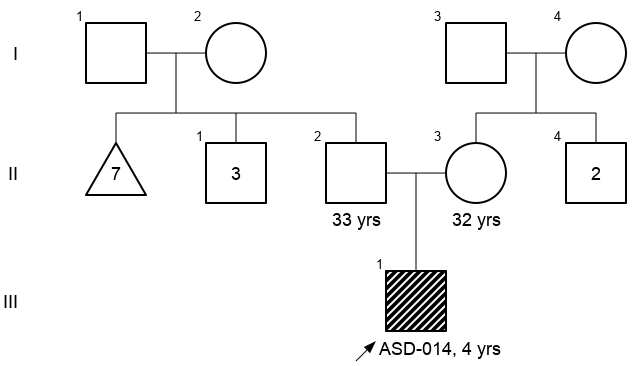 |
| ASD-015  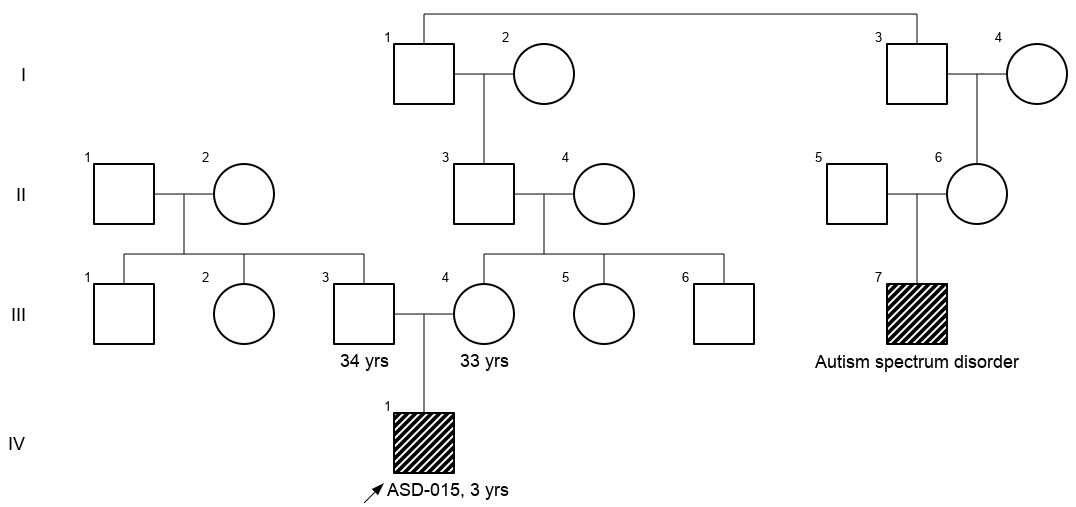 | ASD-016  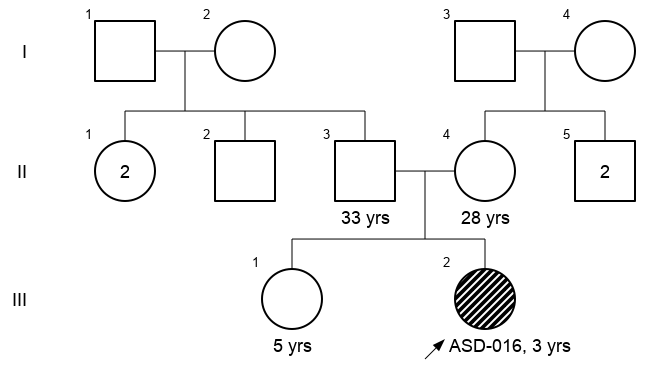 |
| ASD-017  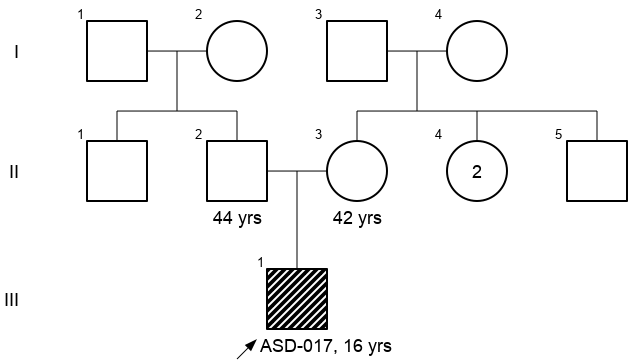 | ASD-018  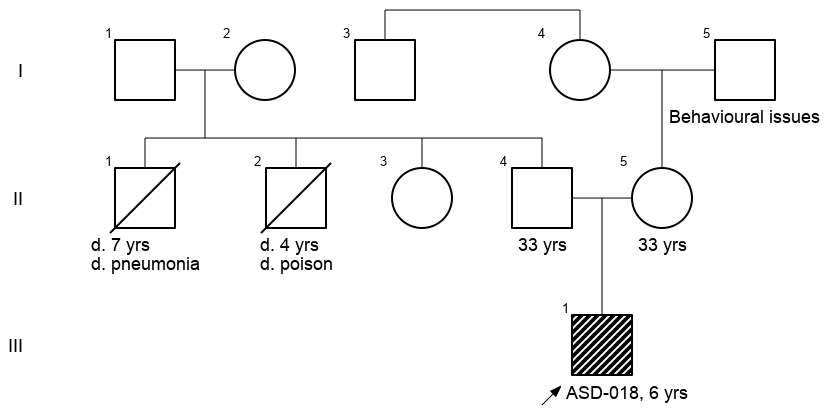 |
| ASD-019  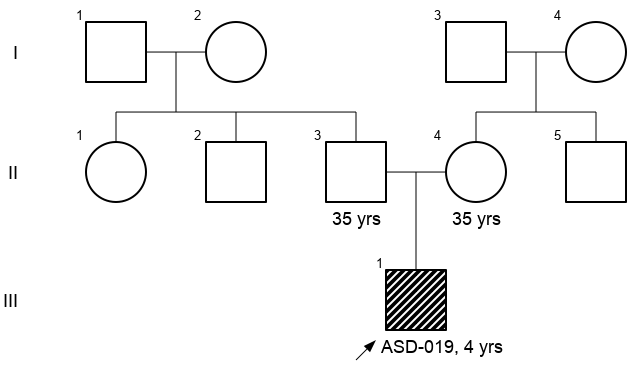 | ASD-020  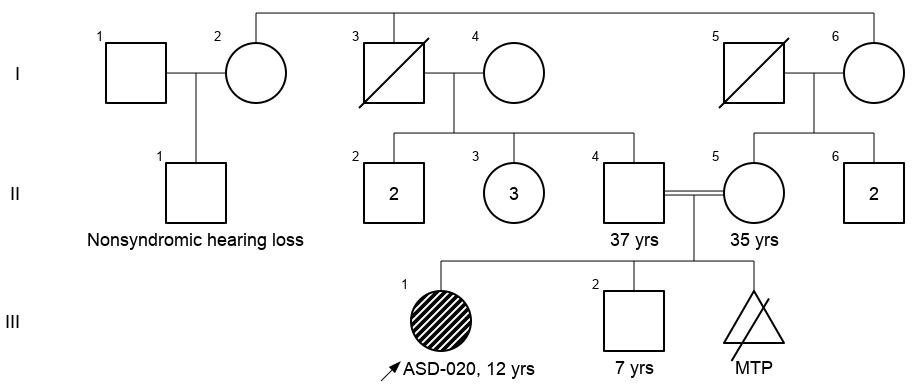 |
| ASD-021  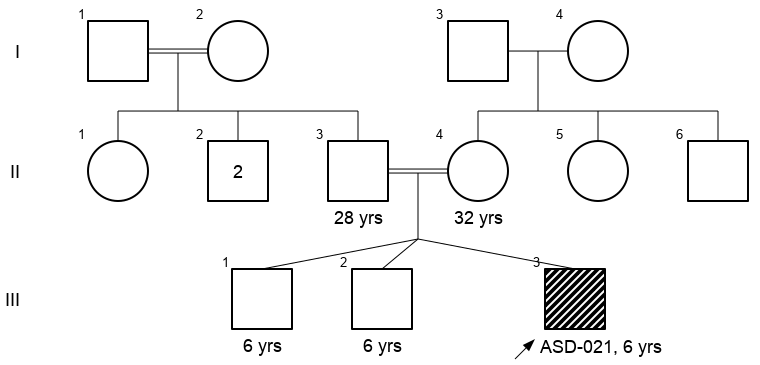 | ASD-022  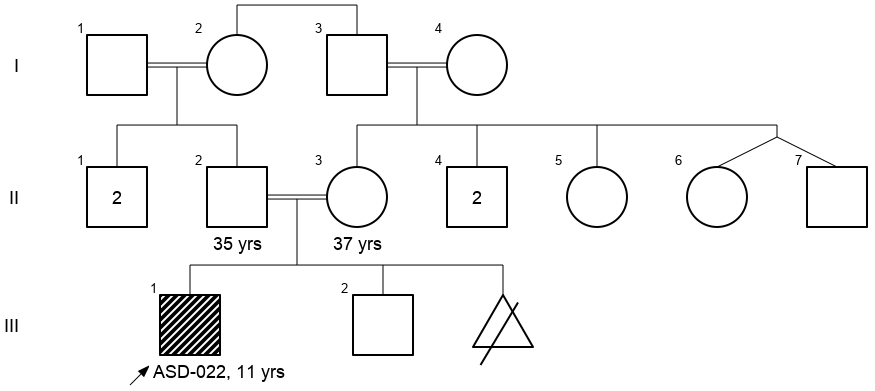 |
| ASD-023  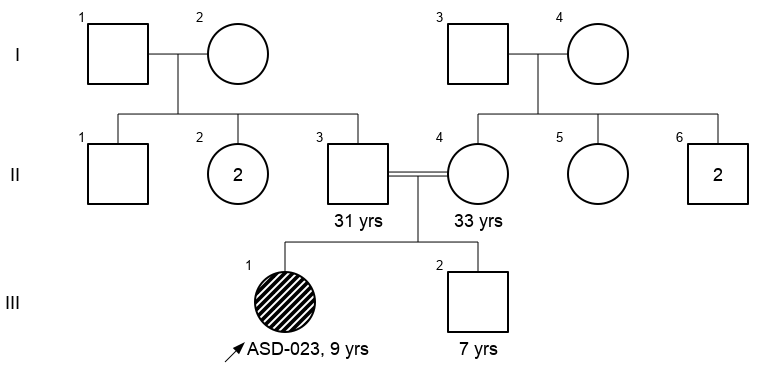 | ASD-024  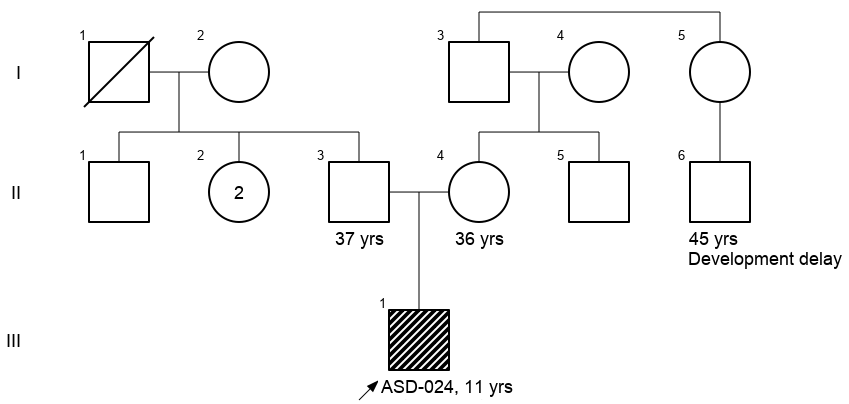 |
| ASD-025  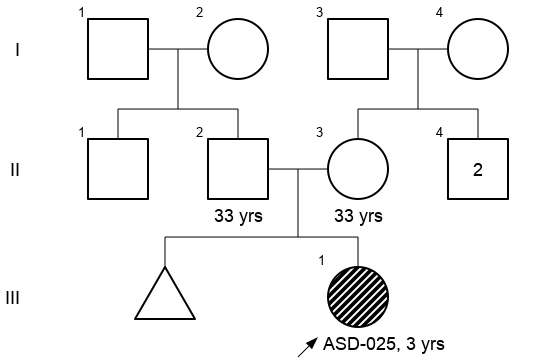 | ASD-026  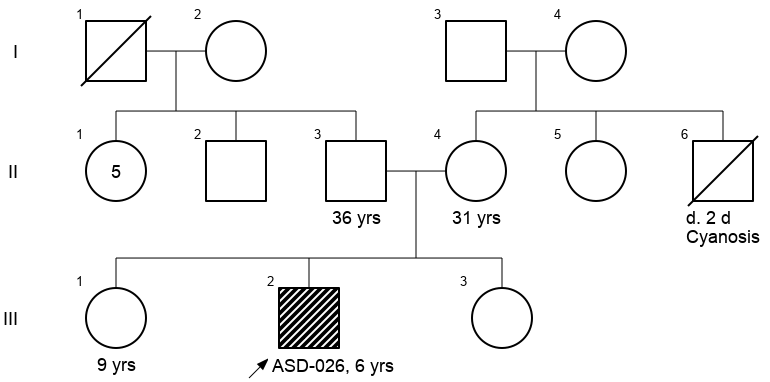 |
| ASD-027  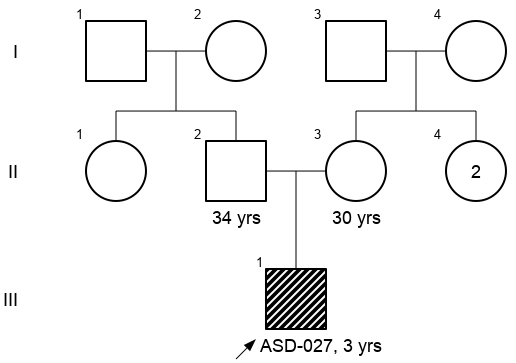 | ASD-028  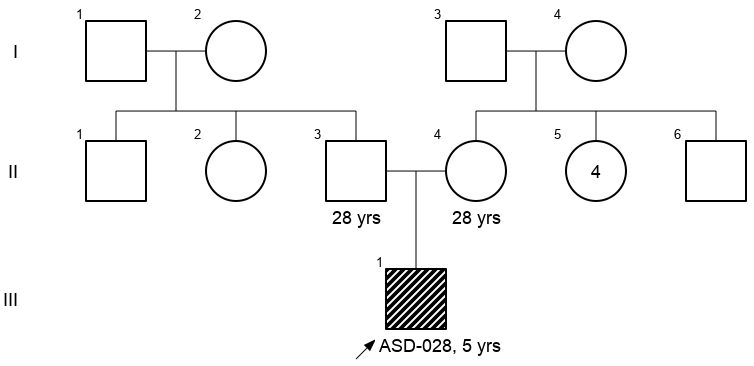 |
| ASD-029  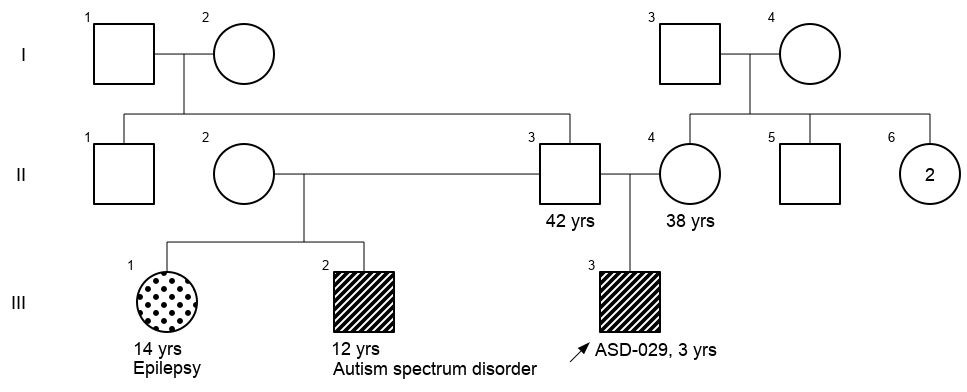 | ASD-030  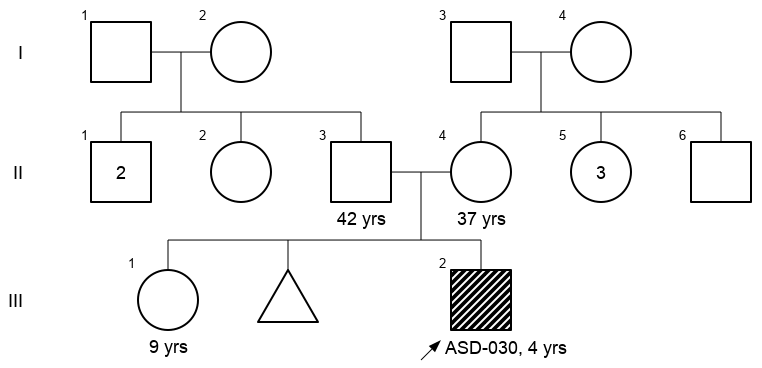 |
| ASD-031  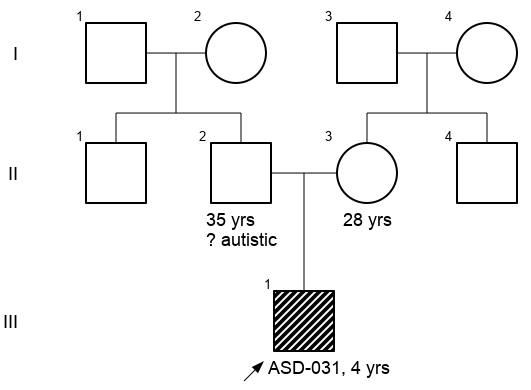 | ASD-032  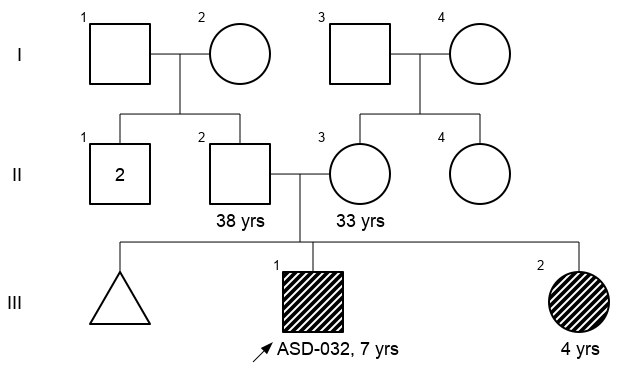 |
| ASD-033  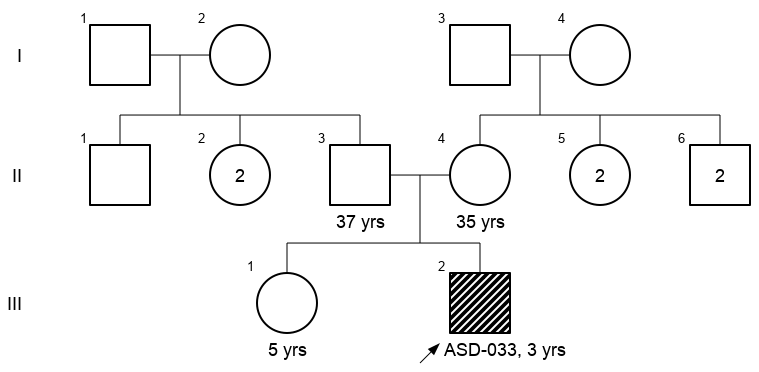 | ASD-034  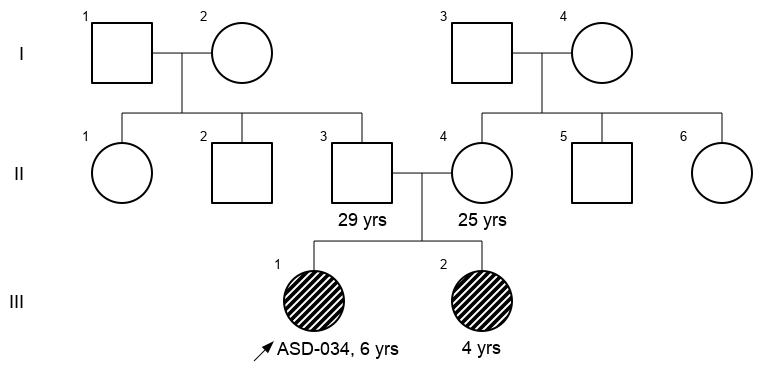 |
| ASD-035  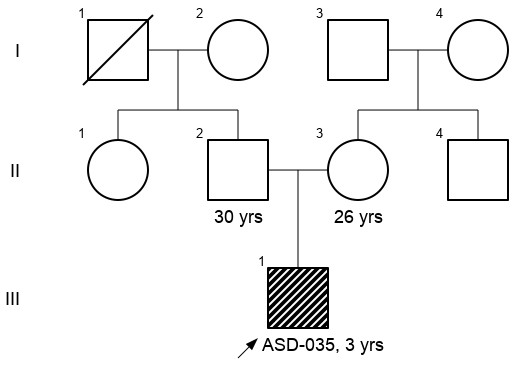 | ASD-036  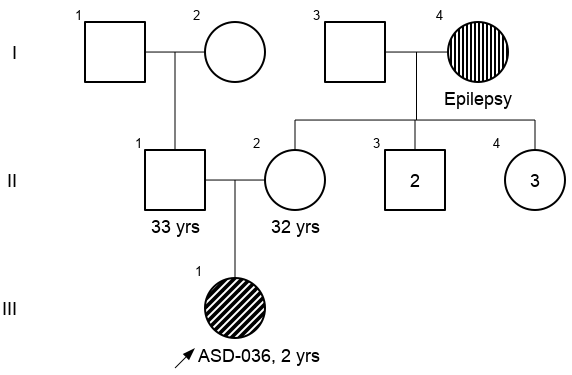 |
| ASD-037  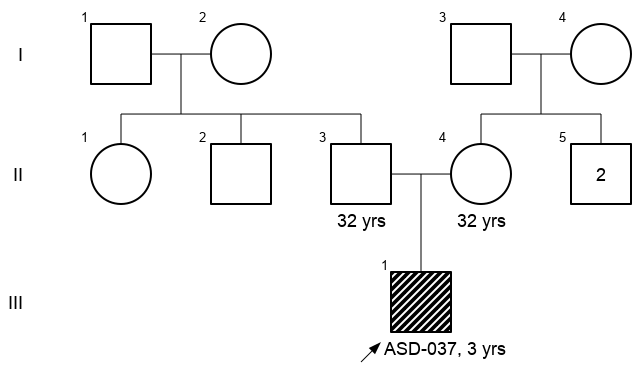 | ASD-039  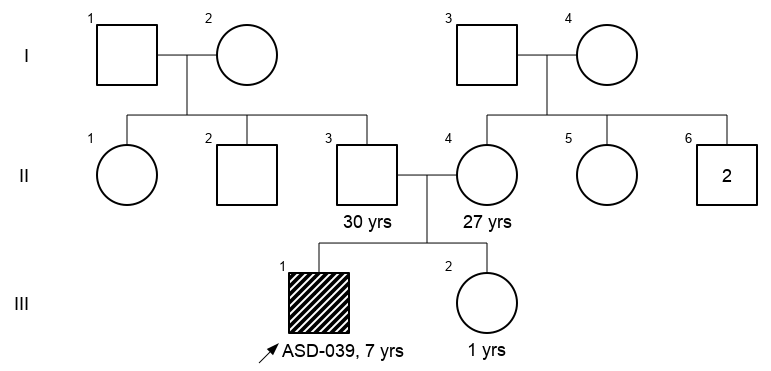 |
| ASD-040  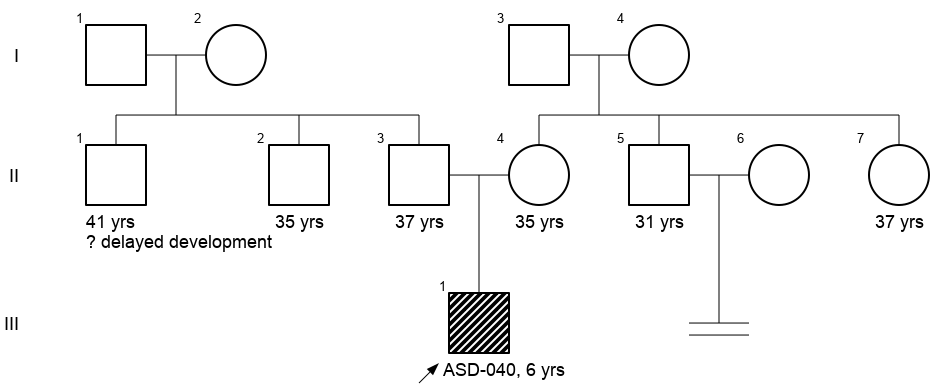 | ASD-041  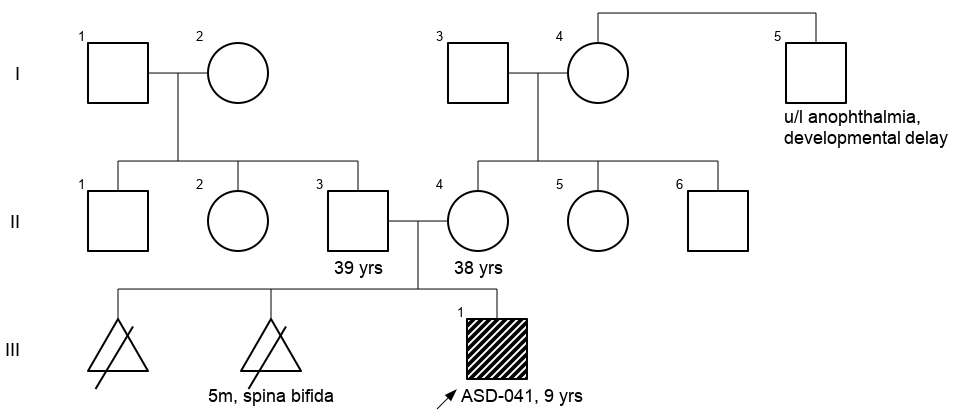 |
| ASD-042  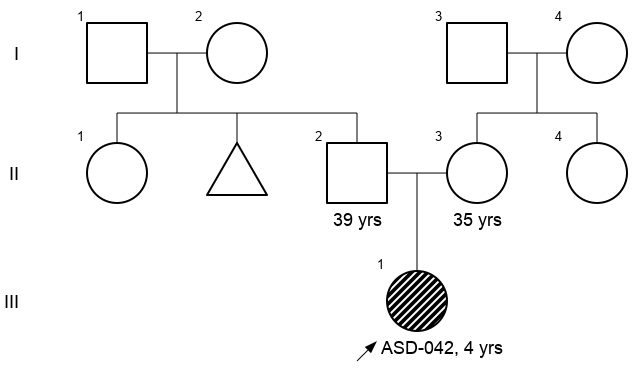 | ASD-043  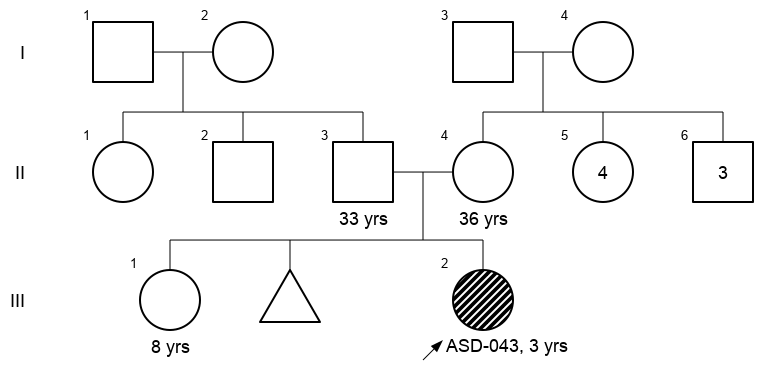 |
| ASD-044  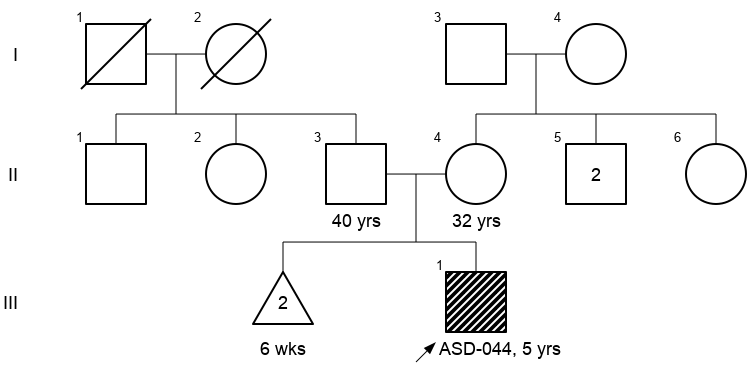 | ASD-045  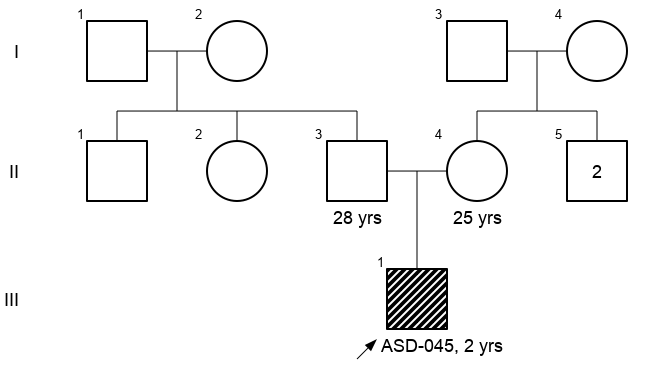 |
| ASD-046  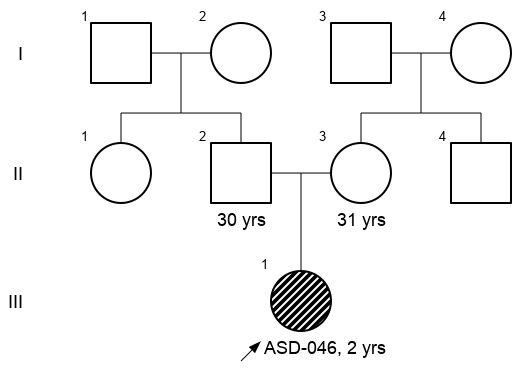 | ASD-047  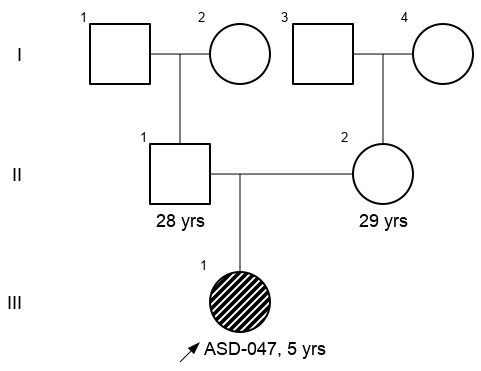 |
| ASD-048  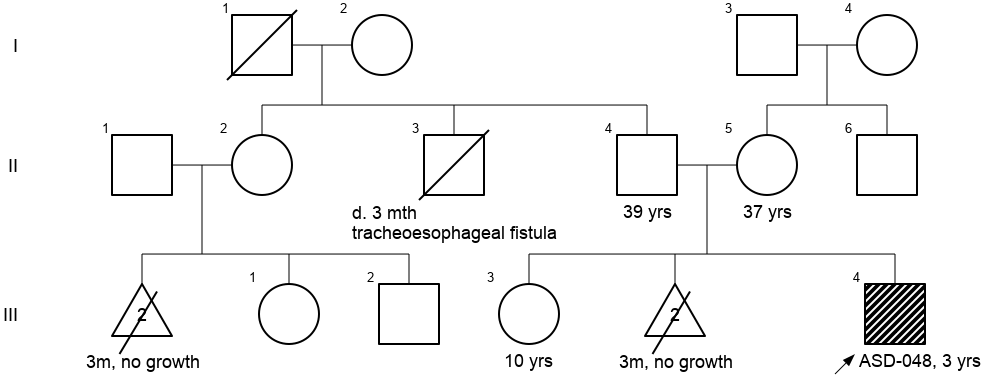 | ASD-049  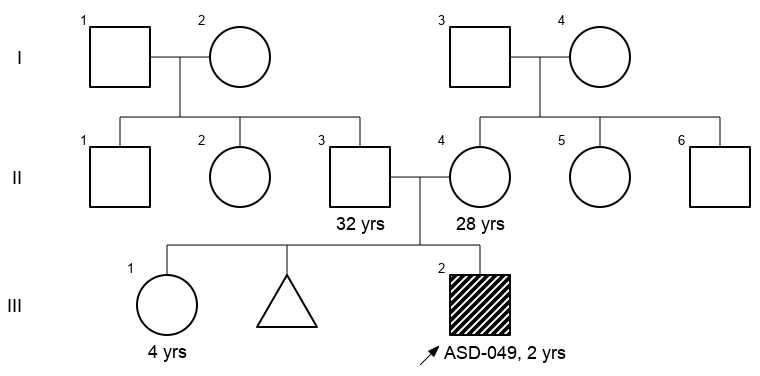 |
| ASD-050  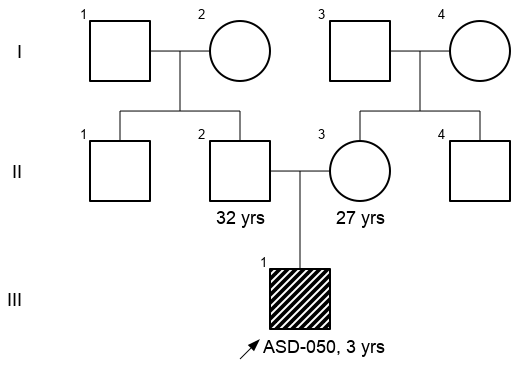 | ASD-051  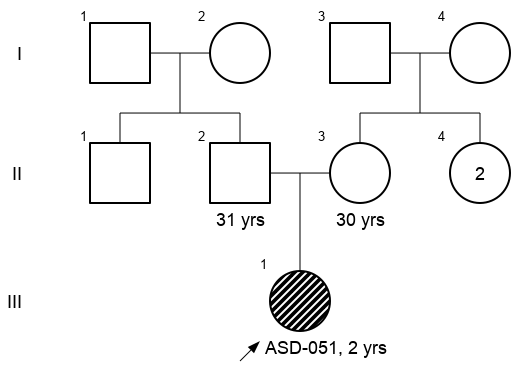 |
| ASD-052  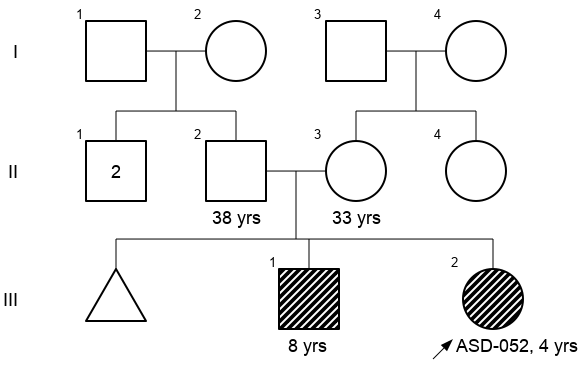 | ASD-053  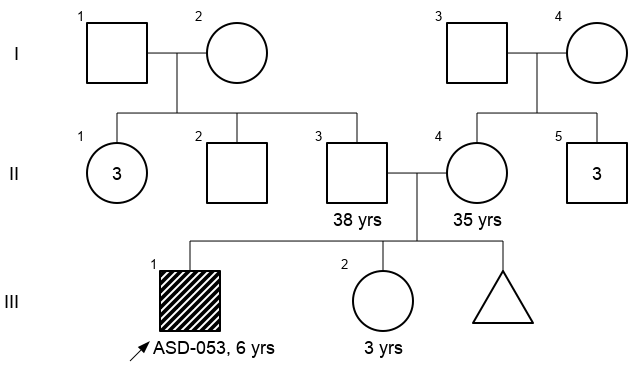 |
| ASD-054  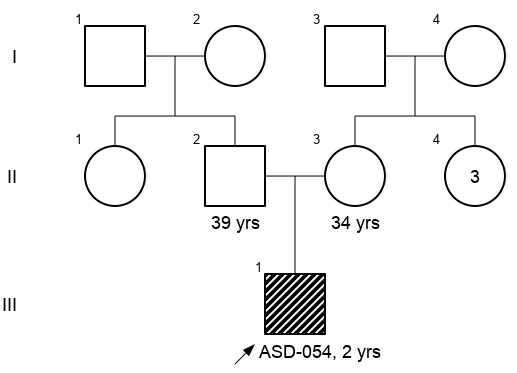 | ASD-055  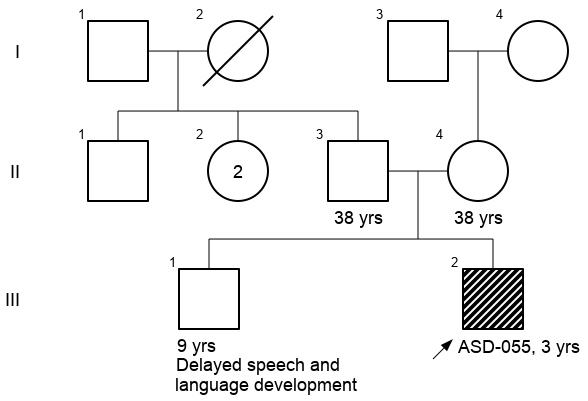 |
| ASD-056  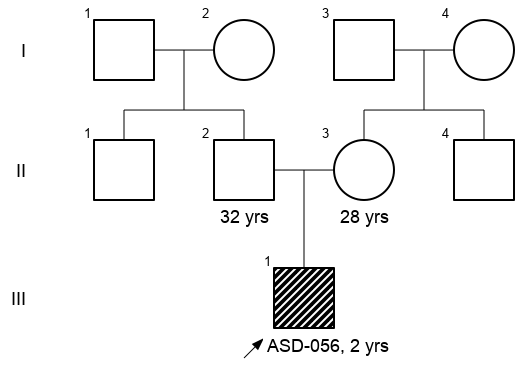 | ASD–057  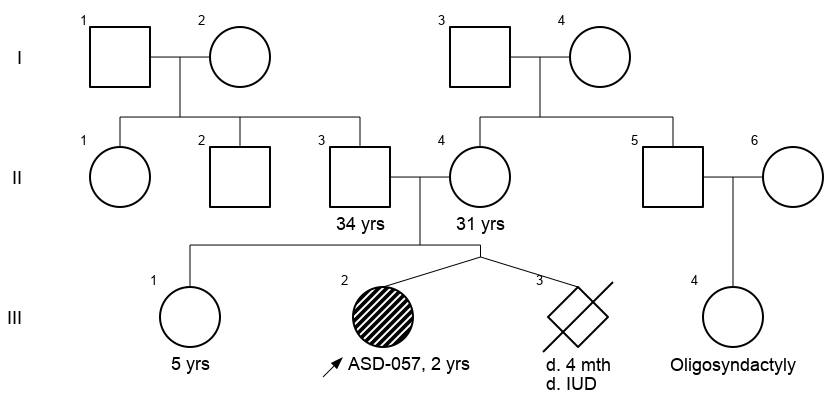 |
| ASD-058  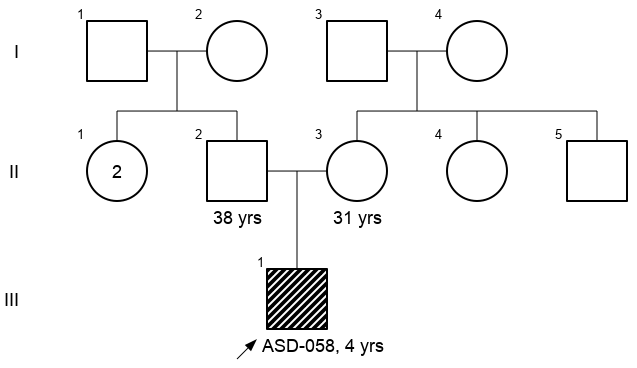 | ASD-059  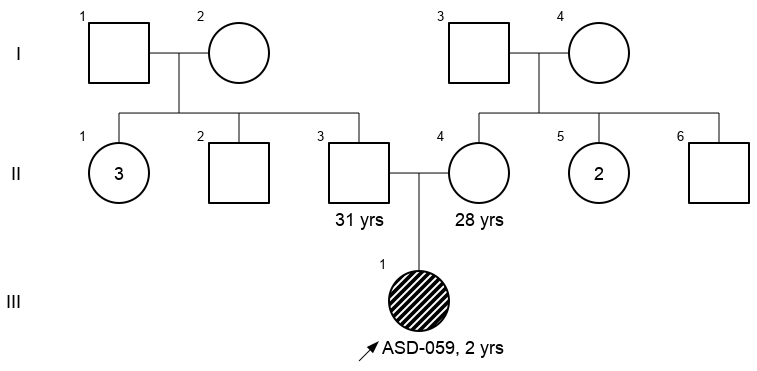 |
| ASD-060  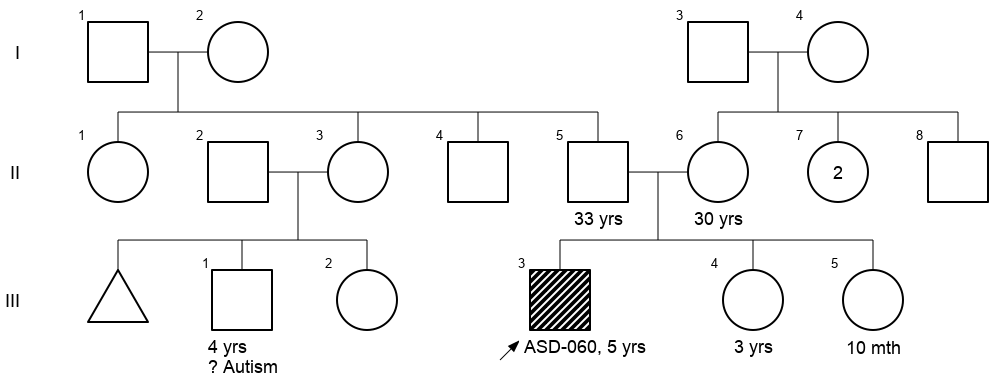 | ASD-061  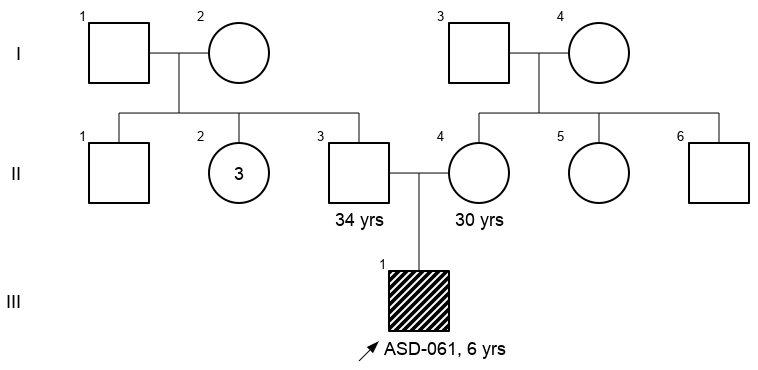 |
| ASD-062  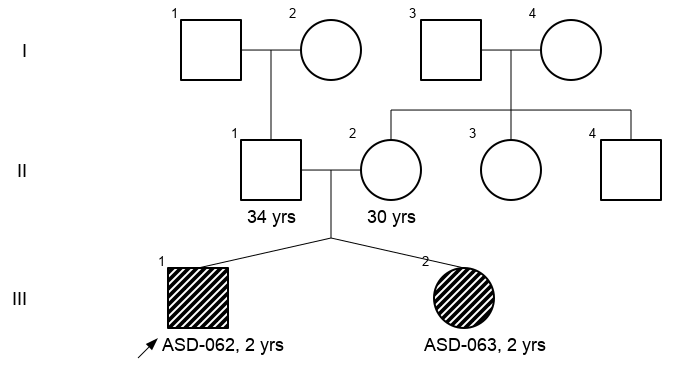 | ASD-063  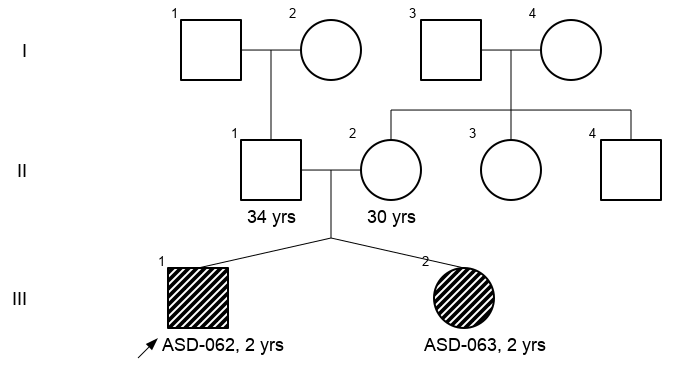 |
| ASD-064  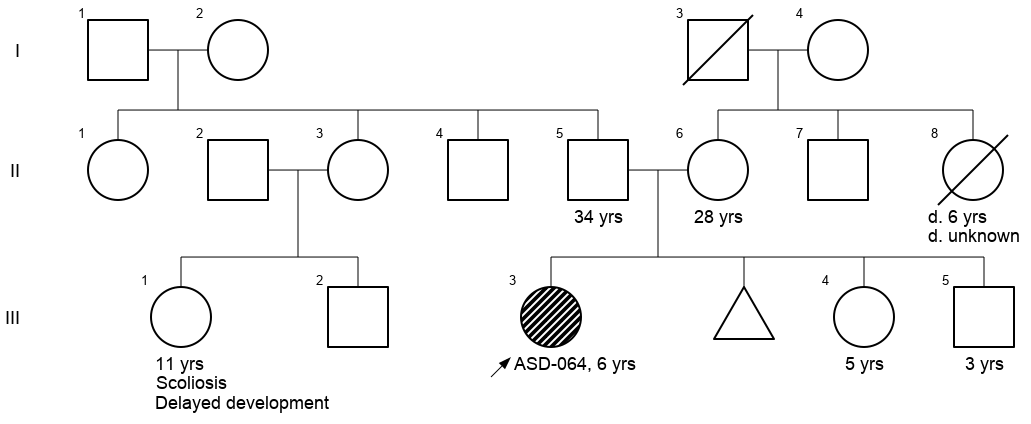 | ASD-065  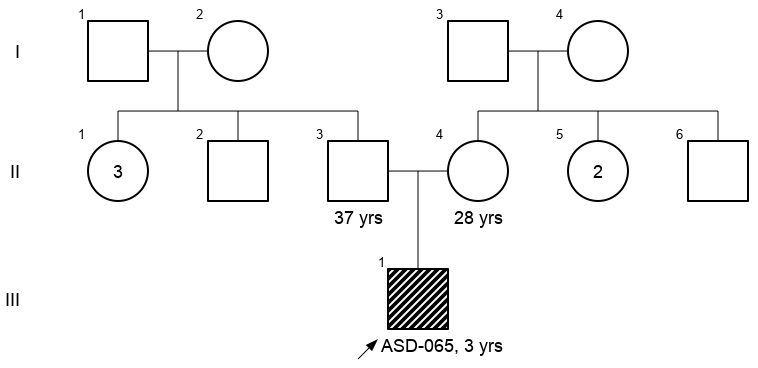 |
| ASD-066  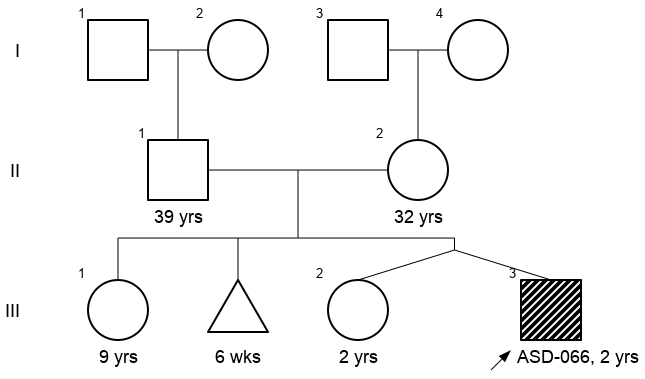 | ASD-067  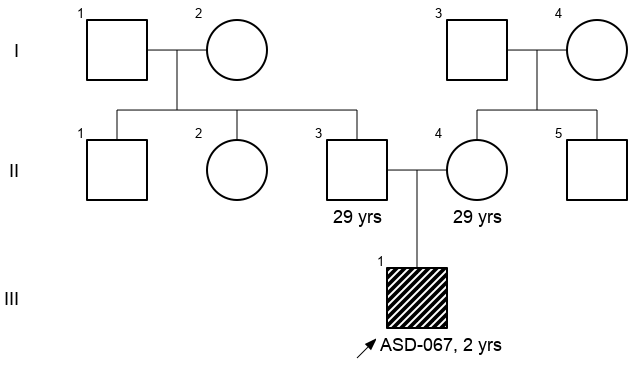 |
| ASD-068  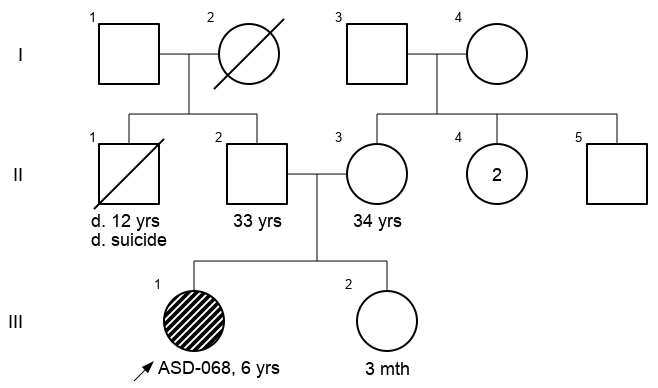 | ASD-069  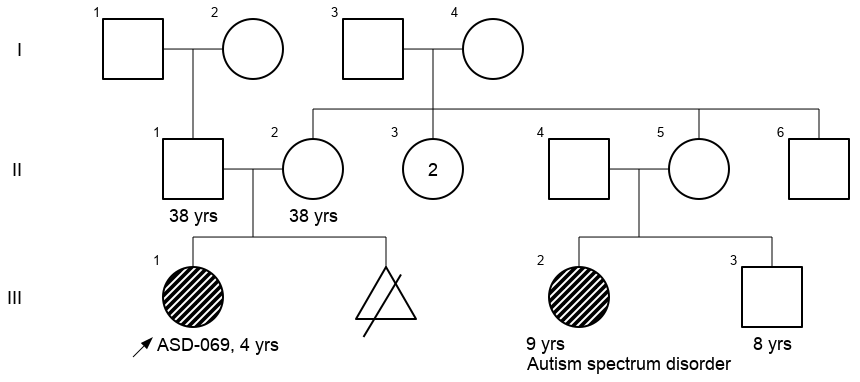 |
| ASD-070  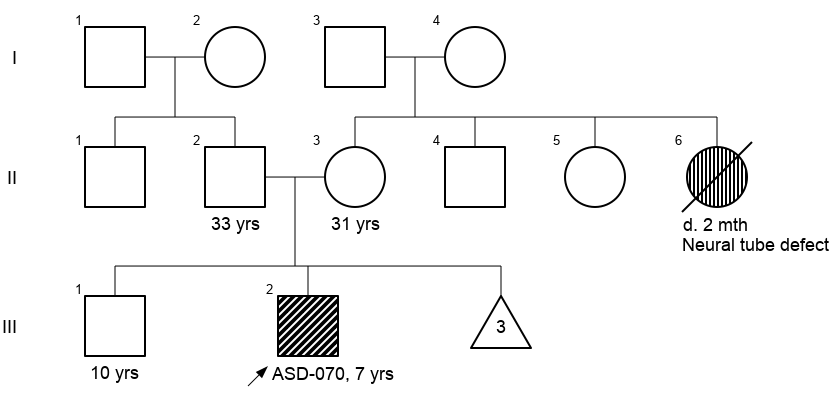 | ASD-071  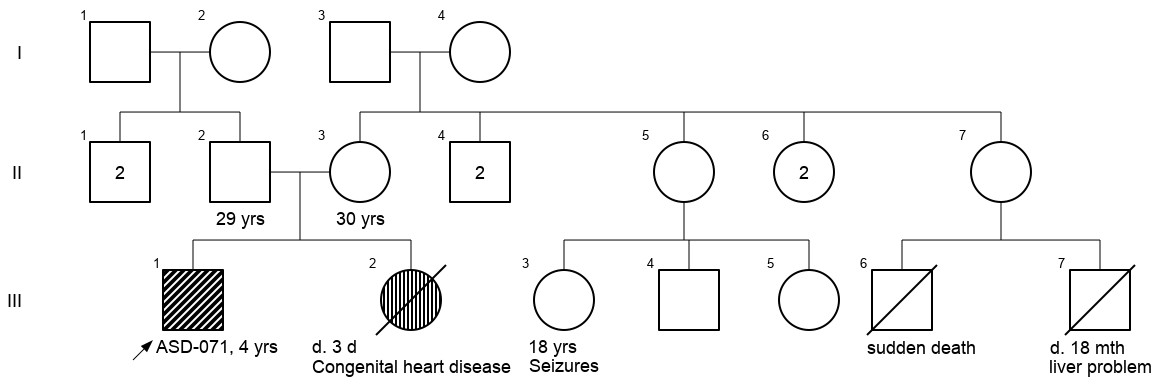 |
| ASD-072  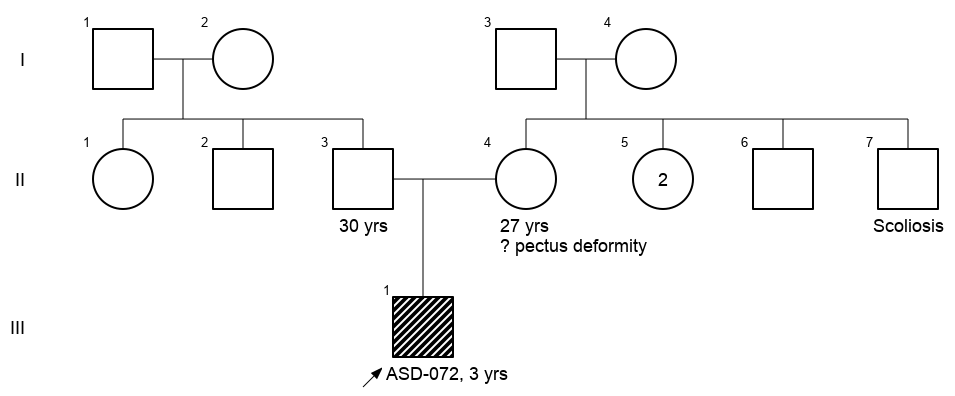 | ASD-073  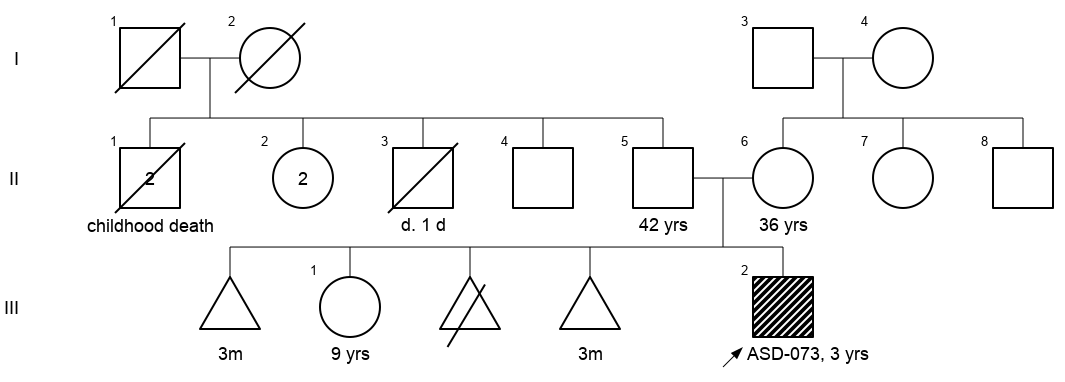 |
| ASD-074  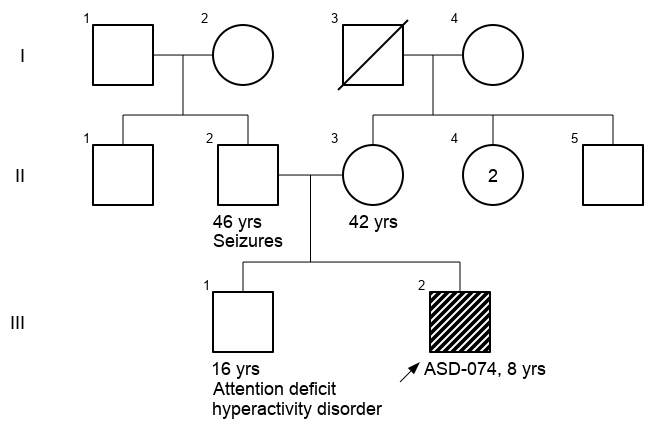 | ASD-075  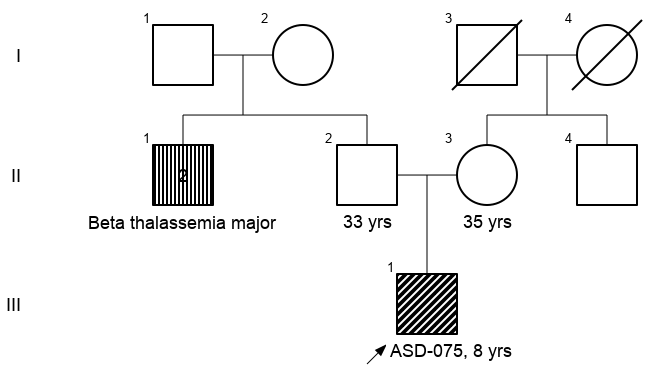 |
| ASD-076  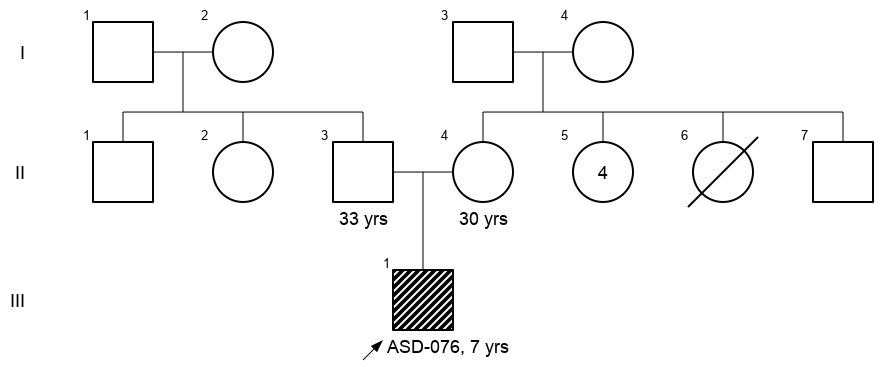 | ASD-077  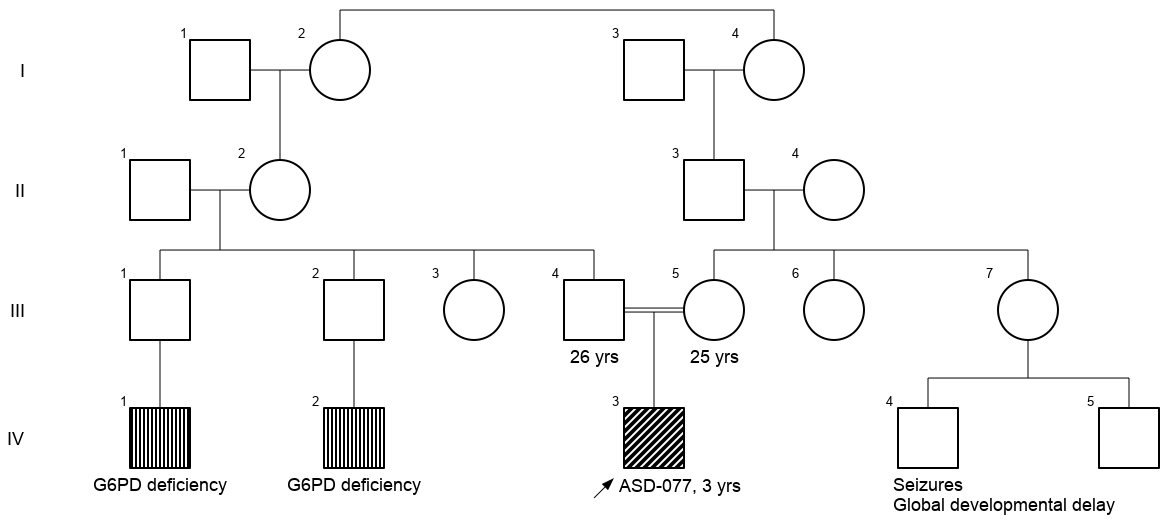 |
| ASD-078  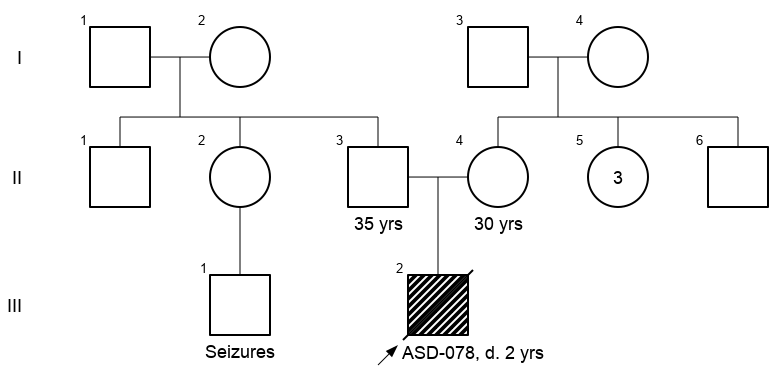 | ASD-079  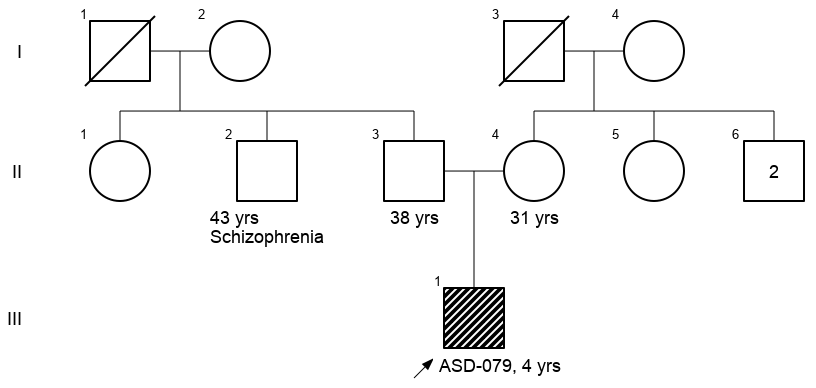 |
| ASD-080  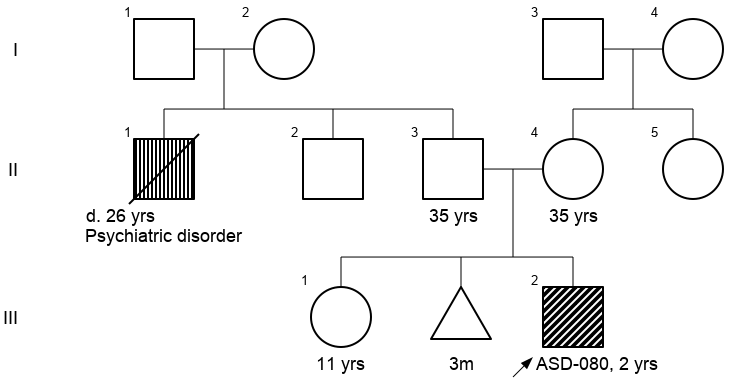 | ASD-081  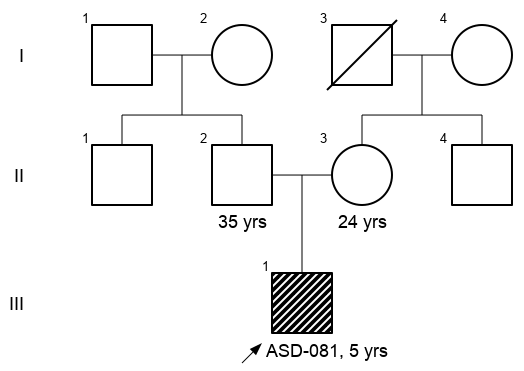 |
| ASD-082  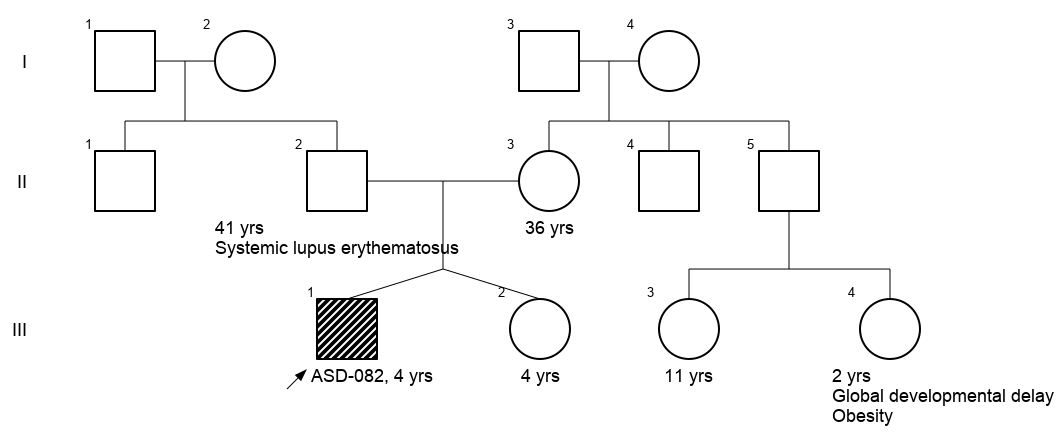 | ASD-083  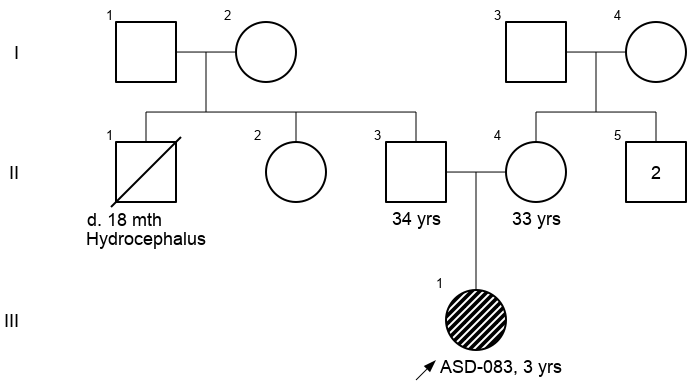 |
| ASD-084  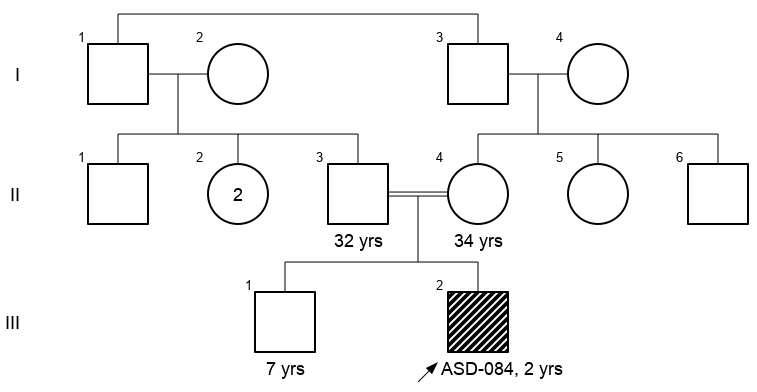 | ASD-085  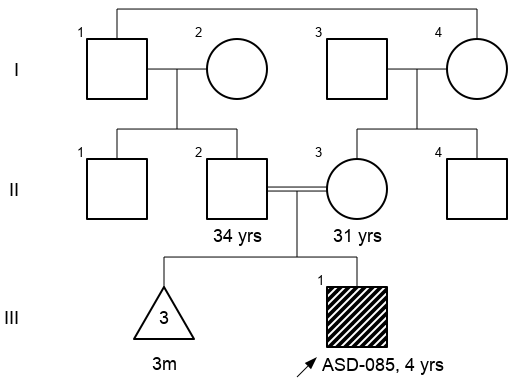 |
| ASD-086  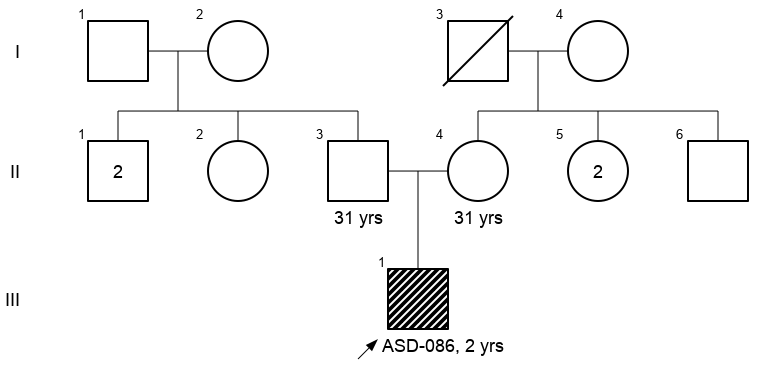 | ASD-087  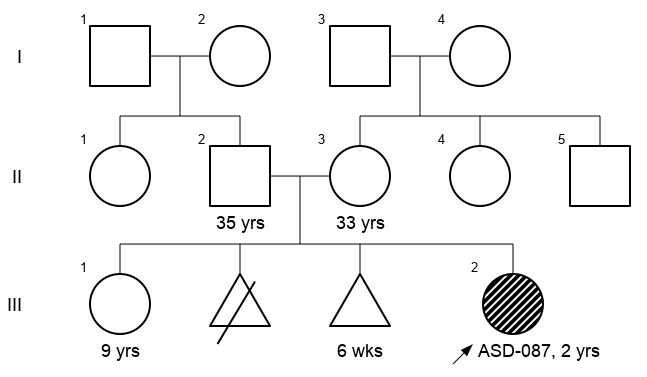 |
| ASD-088  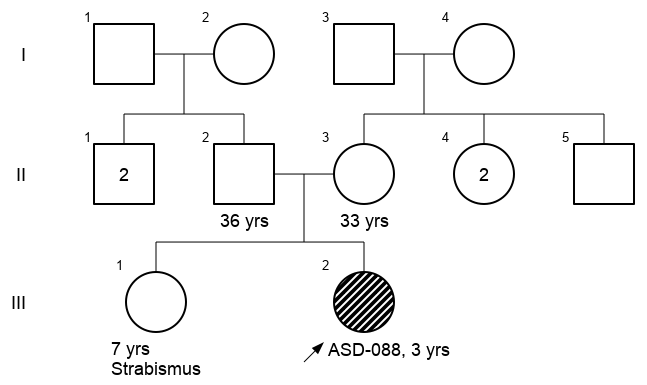 | ASD-089  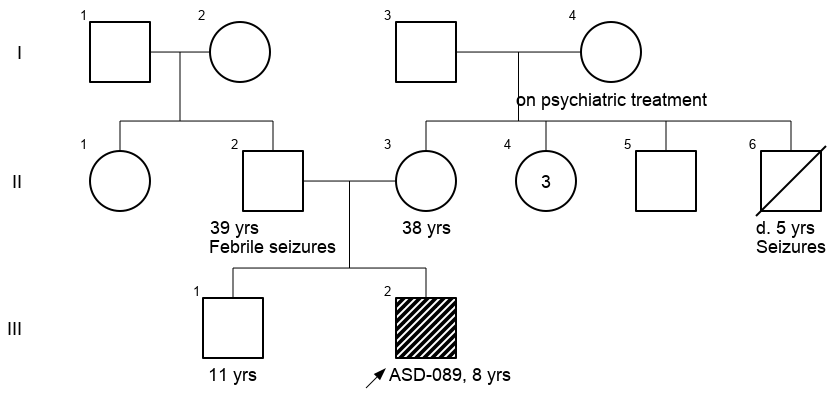 |
| ASD-090  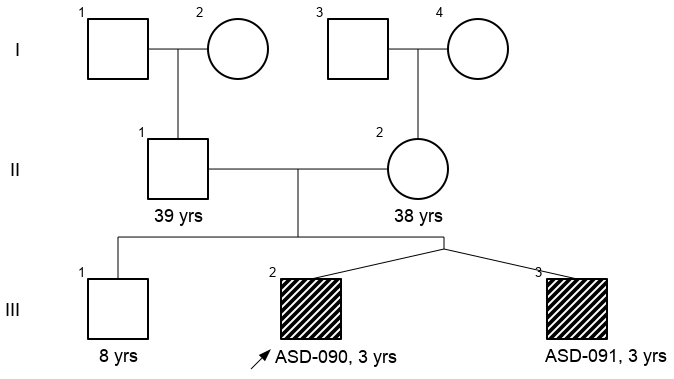 | ASD-091  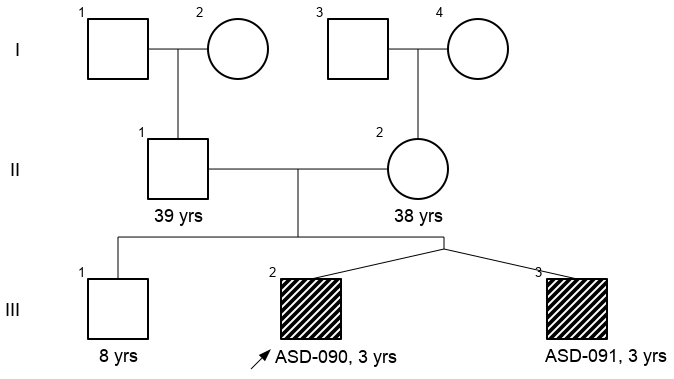 |
| ASD-092  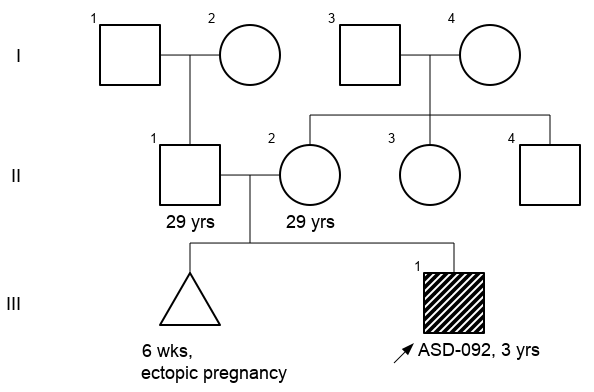 | ASD-093  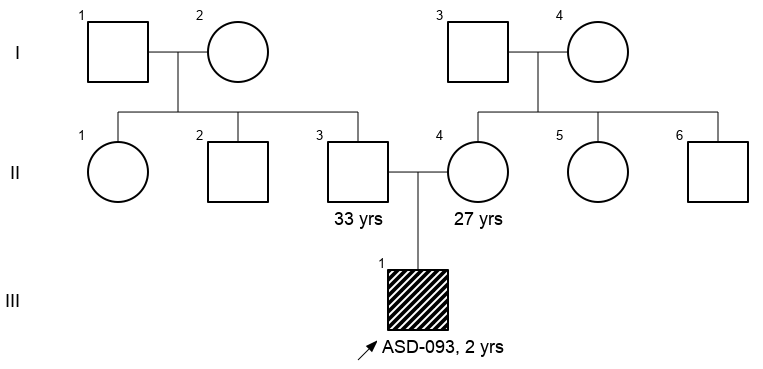 |
| ASD-094  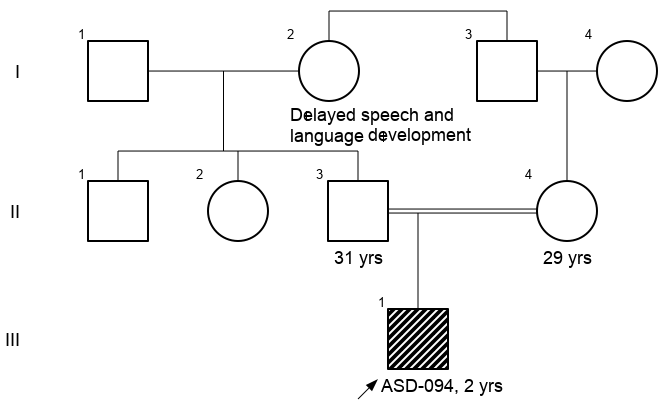 | ASD-095  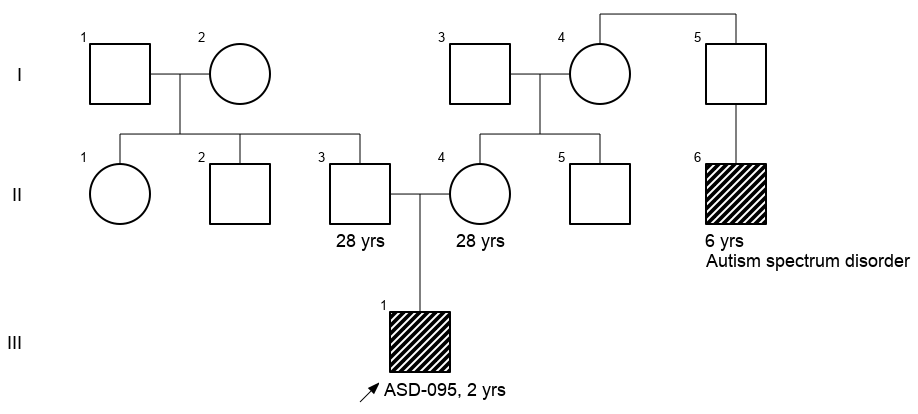 |
| ASD-096  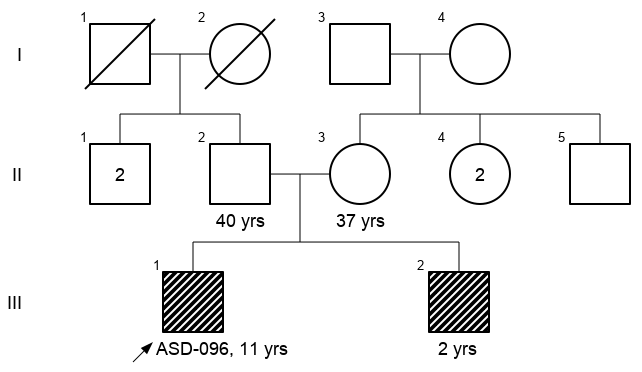 | ASD-097  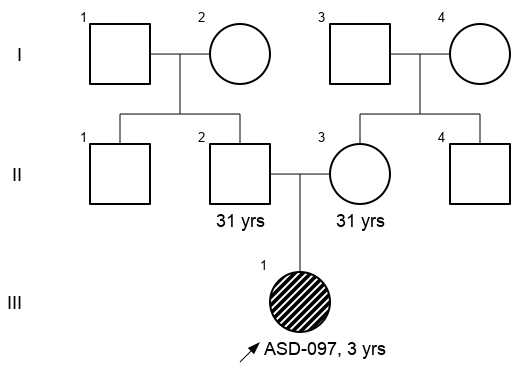 |
| ASD-098  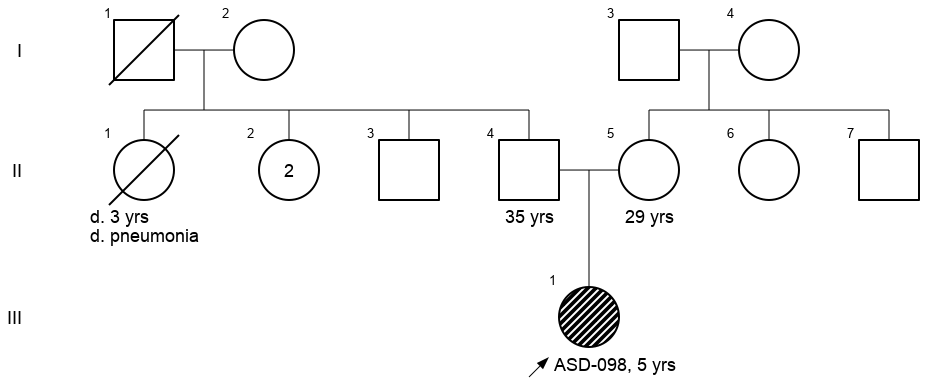 | ASD-099  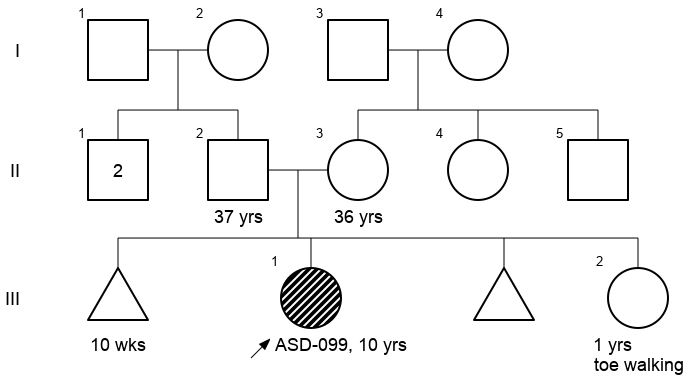 |
| ASD-100  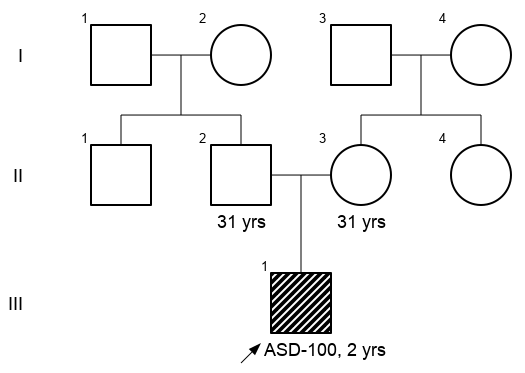 | ASD-101  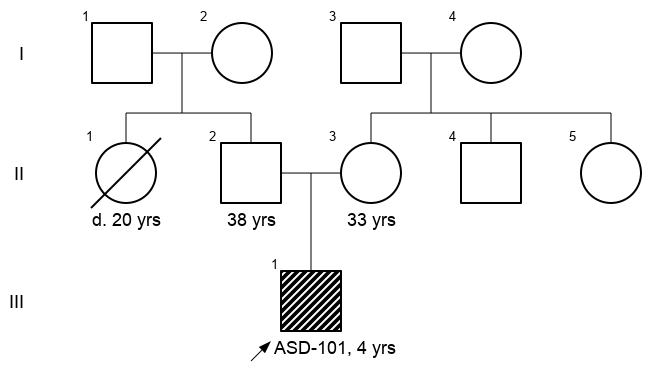 |
| ASD-103 | ASD-104 |
| ASD-105 |  |
