# Supplementary material for: Comparative yield of molecular diagnostic algorithms for autism spectrum disorder diagnosis in India: evidence supporting whole exome sequencing as first tier test
Source: BMC Neurol. 2023 Aug 5;23:292. doi: 10.1186/s12883-023-03341-0 (PMC10403833; doi:10.1186/s12883-023-03341-0)
Supplement: Supplementary file 5 — Supplementary Material 5 [file 12883_2023_3341_MOESM5_ESM.docx]

**Supplementary Table 4**: List of cases with variants of uncertain significance (SNVs) detected using whole exome sequencing.

| **Sr No** | **Case ID** | **Chr No** | **Genomic coordinate** | **Ref allele** | **Alt allele** | **Gene**  **(OMIM ID)** | **Transcript** | **Exon** | **Variant** | **Protein change** | **Zygosity** | **Mode of Inheritance** | **Variant inheritance** | **ACMG-AMP variant classification** | **OMIM Disease**  **(OMIM ID)** |
| --- | --- | --- | --- | --- | --- | --- | --- | --- | --- | --- | --- | --- | --- | --- | --- |
| 01 | ASD-005 | 2 | 162273205 | A | G | *TBR1*  (*604616) | ENST00000389554.3 NM_006593.4 | 1 | c.284A>G | p.His95Arg | Het | AD | Paternal | VUS | Intellectual developmental disorder with autism and speech delay  (#606053) |
| 02 | ASD-007 | X | 46502767 | C | T | *SLC9A7*  (*300368) | ENST00000328306.4 NM_032591.3 | 12 | c.1517G>A | p.Arg506His | Hemi | XLR | Maternal | VUS | Intellectual developmental disorder, X-linked 108  (#301024) |
| 03 | ASD-026 | X | 38240664 | G | A | *OTC*  (*300461) | ENST00000039007.4 NM_000531.6 | 4 | c.368G>A | p.Ser123Asn | Hemi | XL | Maternal | VUS | Ornithine transcarbamylase deficiency  (#311250) |
| 04 | ASD-036 | 7 | 151871299 | C | T | *KMT2C*  (*606833) | ENST00000262189.6 NM_170606.3 | 39 | c.9291G>A | p.Met3097Ile | Het | AD | Maternal | VUS | Kleefstra syndrome 2  (#617768) |
| 05 | ASD-037 | 19 | 13318747 | G | C | *CACNA1A*  (*617106) | ENST00000360228.5 NM_001127222.2 | 47 | c.6901C>G | p.Pro2301Ala | Het | AD | Maternal | VUS | Developmental and epileptic encephalopathy 42  (#601011) |
| 06 | ASD-041 | 19 | 17785548 | C | T | *UNC13A*  (*609894) | ENST00000519716.2 NM_00180421.2 | 3 | c.70G>A | p.Val24Met | Het | UNK | Paternal | VUS | - |
| 07 | ASD-048 | 2 | 166246042 | C | T | *SCN2A*  (*182390) | ENST00000375437.2 NM_001040142.2 | 27 | c.5726C>T | p.Ala1909Val | Het | AD | Maternal | VUS | Developmental and epileptic encephalopathy 11  (#613721) |
| 08 | ASD-051 | 17 | 7106862 | G | A | *DLG4*  (*602887) | ENST00000399510.2 NM_001365.4 | 8 | c.515C>T | p.Thr172Ile | Het | AD | Maternal | VUS | Intellectual developmental disorder, autosomal dominant 62  (#618793) |
| 09 | ASD-055 | 15 | 28493730 | C | T | *HERC2*  (*605837) | ENST00000261609.7 NM_004667.6 | 21 | c.3203G>A | p.Gly1068Glu | Comp het | AR | Maternal | VUS | Intellectual developmental disorder, autosomal recessive 38  (#615516) |
|  |  |  | 28459263 | G | A |  |  | 41 | c.6514C>T | p.Arg2172Cys |  |  | Paternal | VUS |  |
| 10 | ASD-060 | 5 | 45303859 | A | G | *HCN1*  (*618482) | ENST00000303230.4 NM_021072.4 | 6 | c.1460T>C | p.Met487Thr | Het | AD | Maternal | VUS | Generalized epilepsy with febrile seizures plus, type 10  (#602780) |
| 11 | ASD-066 | 7 | 151845739 | C | T | *KMT2C*  (*606833) | ENST00000262189.6 NM_170606.3 | 52 | c.13273G>A | p.Asp4425Asn | Het | AD | Paternal | VUS | Kleefstra syndrome 2  (#617768) |
| 12 | ASD-069 | 17 | 7321003 | C | T | *NLGN2*  (*606479) | ENST00000302926.2 NM_020795.3 | 7 | c.2393C>T | p.Pro798Leu | Het | UNK | Maternal | VUS | - |
| 13 | ASD-071 | 14 | 94089065 | T | C | *UNC79*  (*616884) | ENST00000256339.4 NM_020818.5 | 30 | c.4955T>C | p.Phe1652Ser | Het | UNK | Maternal | VUS | - |
| 14 | ASD-077 | X | 70341264 | C | G | *MED12*  (*300188) | ENST00000333646.6 NM_005120.3 | 6 | c.823C>G | p.Leu275Val | Hemi | XL | Maternal | VUS | Ohdo syndrome, X-linked  (#300895) |
| 15 | ASD-078 | X | 53458478 | T | G | *HSD17B10*  (*300256) | ENST00000168216.6 NM_004493.3 | 6 | c.660A>C | p.Gln220His | Hemi | XL | Maternal | VUS | HSD10 mitochondrial disease  (#300438) |
| 16 | ASD-089 | X | 153219870 | G | A | *HCFC1*  (*300019) | ENST00000310441.7 NM_005334.3 | 17 | c.3980C>T | p.Thr1327Met | Hemi | XLR | Maternal | VUS | Methylmalonic aciduria and homocysteinemia, cblX type  (#309541) |
| 17 | ASD-093 | 15 | 93499699 | G | A | *CHD2*  (*602119) | ENST00000394196.4 NM_001271.4 | 16 | c.1820G>A | p.Gly607Asp | Het | AD | Paternal | VUS | Developmental and epileptic encephalopathy 94  (#615369) |
| 18 | ASD-094 | 6 | 170593031 | C | T | *DLL1*  (*606582) | ENST00000366756.3 NM_005618.4 | 9 | c.1336G>A | p.Aso446Asn | Het | AD | Paternal | VUS | Neurodevelopmental disorder with nonspecific brain abnormalities and with or without seizures  (#618709) |
| 19 | ASD-095 | 1 | 155491082 | C | T | *ASH1L*  (*607999) | ENST00000392403.3 NM_018489.3 | 2 | c.229G>A | p.Glu77Lys | Het | AD | Paternal | VUS | Intellectual developmental disorder, autosomal dominant 52  (#617796) |
| 20 | ASD-097 | 16 | 30745917 | A | C | *SRCAP*  (*136140) | ENST00000262518.4 NM_006662.3 | 31 | c.6710A>C | p.Gln2237Pro | Het | AD | Paternal | VUS | Developmental delay, hypotonia, musculoskeletal defects, and behavioral abnormalities  (#619595) |
| 21 | ASD-098 | 7 | 151902250 | G | A | *KMT2C*  (*606833) | ENST00000262189.6 NM_170606.3 | 25 | c.3902C>T | p.Ser1301Phe | Het | AD | Paternal | VUS | Kleefstra syndrome 2  (#617768) |

Het: heterozygous; Hom: homozygous; Hemi: hemizygous; Comp het: Compound heterozygous; AD: autosomal dominant; AR: autosomal recessive; XL: X-linked; UNK: unknown
